# Etiologies and comorbidities of meningitis deaths in children under 5 years in high-mortality settings: Insights from the CHAMPS Network in the post-pneumococcal vaccine era

**DOI:** 10.1016/j.jinf.2024.106341

**Published:** 2024-12

**Authors:** Sana Mahtab, Zachary J. Madewell, Vicky Baillie, Ziyaad Dangor, Sanjay G. Lala, Nega Assefa, Mulu Berihun, Lola Madrid, Lemma Demissie Regassa, J. Anthony G. Scott, Soter Ameh, Joseph S. Bangura, Okokon Ita, Erick Kaluma, Ikechukwu Udo Ogbuanu, Brigitte Gaume, Karen L. Kotloff, Samba O. Sow, Milagritos D. Tapia, Sara Ajanovic, Marcelino Garrine, Inacio Mandomando, Rosauro Varo, Elisio G. Xerinda, Muntasir Alam, Shams El Arifeen, Emily S. Gurley, Mohammad Zahid Hossain, Afruna Rahman, Victor Akelo, Kitiezo Aggrey Igunza, Clayton Onyango, Dickens Onyango, Jennifer R. Verani, Portia Mutevedzi, Cynthia G. Whitney, Dianna M. Blau, Shabir A. Madhi, Quique Bassat, CHAMPSconsortium

**Affiliations:** a South African Medical Research Council Vaccines and Infectious Diseases Analytics Research Unit, University of the Witwatersrand, Johannesburg, South Africa; b Global Health Center, Centers for Disease Control and Prevention, Atlanta, GA, USA; c Department of Paediatrics & Child Health, Faculty of Health Sciences, University of the Witwatersrand, South Africa; d College of Health and Medical Sciences, Haramaya University, Harar, Ethiopia; e Department of Infectious Disease Epidemiology, London School of Hygiene & Tropical Medicine, London, United Kingdom; f CHAMPS Sierra Leone, Freetown, Sierra Leone; g Ministry of Health and Sanitation, Freetown, Sierra Leone; h University of Calabar Teaching Hospital, Nigeria; i Center for Vaccine Development and Global Health, University of Maryland School of Medicine, Baltimore, MD, USA; j Centre pour le Développement des Vaccins, Ministère de la Santé, Bamako, Mali; k Centro de Investigação em Saúde de Manhiça, Maputo, Mozambique; l ISGlobal, Barcelona, Spain, Facultat de Medicina i Ciències de la Salut, Unviersitat de Barcelona (UB), Barcelona, Spain; m Instituto Nacional de Saúde, Ministério de Saúde, Maputo, Mozambique; n International Centre for Diarrhoeal Disease Research Bangladesh (ICDDRB), Dhaka, Bangladesh; o Department of Epidemiology, Johns Hopkins Bloomberg School of Public Health, Baltimore, MD, USA; p Centers for Disease Control and Prevention–Kenya, Kisumu, Kenya; q Kenya Medical Research Institute, Center for Global Health Research, Kisumu, Kenya; r Center for Global Health, US Centers for Disease Control and Prevention, Nairobi, Kenya; s Kisumu County Department of Health, Kisumu, Kenya; t National Center for Immunization and Respiratory Diseases, US Centers for Disease Control and Prevention, Atlanta, GA, USA; u Emory Global Health Institute, Emory University, Atlanta, GA, USA; v Wits Infectious Diseases and Oncology Research Institute, Faculty of Health Sciences, University of the Witwatersrand, Johannesburg, South Africa; w ICREA, Pg. Lluís Companys 23, 08010 Barcelona, Spain; x Pediatrics Department, Hospital Sant Joan de Déu, Universitat de Barcelona, Esplugues, Barcelona, Spain; y Consorcio de Investigación Biomédica en Red de Epidemiología y Salud Pública–CIBERESP, Madrid, Spain

**Keywords:** Meningitis, Minimally invasive tissue sampling, Pathogens, Occurred ≥72 h post-hospital admission, Community associated meningitis

## Abstract

**Background:**

The role of meningitis in causing deaths and in children under 5 is unclear, especially since widespread use of vaccines to prevent common causes of meningitis. Child Health and Mortality Prevention Surveillance (CHAMPS) uses post-mortem minimally invasive tissue sampling (MITS) and ante-mortem data to explore death causes. We aimed to assess meningitis’s contribution to mortality and identify causative pathogens in children under 5 within CHAMPS Network sites**.**

**Method:**

In this observational study, we analyzed deaths in live-born children <5 years of age that occurred between December 16, 2016, and December 31, 2023, in CHAMPS catchments in six sub-Saharan African countries (Ethiopia, Kenya, Mali, Mozambique, Sierra Leone, South Africa) and Bangladesh. MITS was conducted within 24–72 h of death, including blood and cerebrospinal fluid (CSF) culture, multi-organism targeted nucleic acid amplification tests on blood, CSF and lung tissue, and histopathology of lung, liver and brain. Expert panels at each site reviewed data to attribute causes of death following ICD-10 standards.

**Result:**

Meningitis was in the causal pathway for 7.0% (270/3857) of deaths; in 4.8% (13/270) meningitis was considered the underlying condition. Neonates accounted for 65.9% (178/270) and infants or children 34.1% (92/270). Among neonatal meningitis deaths, 55.6% (99/178) occurred ≥72 h post-hospital admission; and common pathogens were *Acinetobacter baumannii* (49.5%, 49/99; mainly from South Africa) and *Klebsiella pneumoniae* (40.4%, 40/99). Forty-four percent (79/178) of neonatal meningitis deaths were community-associated, primarily due to *K. pneumoniae* (35.4%, 28/79) and *Escherichia coli* (13.9%, 11/79). Among infant and child meningitis deaths, 43.5% (40/92) occurred ≥72 h post-admission; and common pathogens were *K. pneumoniae* (42.5%,17/40) and *A. baumannii* (17.5%, 7/40). Among community-associated meningitis deaths in infants and children (56.5%, 52/92), *Streptococcus pneumoniae* (34.6%, 18/52) and *K. pneumoniae* (19.2%, 10/52) were common pathogens. Pathogen prevalence varied by region.

**Conclusion:**

Our study highlights meningitis as a significant contributor to under-5 mortality in low-middle-income countries. The prominent role of *K. pneumoniae* and *A. baumannii,* particularly in healthcare settings and specific regions, highlights the need for better infection control, targeted interventions, and more effective treatment strategies.

## Highlights

• Meningitis was found to be one of the leading causes of U5 deaths in the CHAMPS network, exceeding the WHO’s estimated rate.
• This discrepancy suggests current estimates may underestimate meningitis cases by not considering all contributing factors.
• High rates of resistant organisms like *K pneumoniae* and *A baumannii* increase mortality risk if antibiotics aren't used.
• Vaccination has reduced *S. pneumoniae* cases, it remains a significant pathogen for community-acquired meningitis.

## Introduction

Despite significant progress in reducing child mortality, approximately five million children still die in 2019, with nearly half within the first 28 days of life.^1^ Ninety-nine percent of under-5 childhood deaths occur in low- and middle-income countries (LMICs), highlighting global health disparities.^2^ The United Nations Sustainable Development Goal (SDG) 3.2 calls to end preventable deaths among newborns and under-5s by 2030, with a target of at least 25 per 1000 live births.^2^ Nevertheless, in 2019, an estimated 38 deaths per 1000 live births were still attributed to preventable and treatable diseases.^2^

Infectious meningitis is a preventable disease with high fatality and neurological sequelae rates, can occur as devastating epidemics, and remains a major public health concern.^3^ In 2019, the Global Burden of Disease report highlighted meningitis as one of the common disease among children under 5, causing 112,000 deaths and 1.28 million incident cases.^4^ Furthermore, meningitis poses a diagnostic challenge due to its nonspecific clinical presentation, which often overlaps with other severe conditions.^5^ Diagnostic methods, such as lumbar puncture to obtain cerebrospinal fluid (CSF), require specialized expertise and infrastructure for analysis, which is frequently unavailable in LMICs.^6^ While the World Health Organization has outlined a global roadmap to defeat meningitis by 2030,^7^ achieving this goal necessitates an accurate understanding of the global burden of meningitis and its causes. The burden and distribution of pathogens causes meningitis deaths has likely shifted significantly in recent years, however, with introduction of vaccines to prevent the most common causes, including against *Haemophilus influenzae* type B (Hib), *Streptococcus pneumoniae*, and *Neisseria meningitidis.* Estimates of the burden of pathogen-specific meningitis and mortality at the global level would enable the identification of more effective use of resources and could illuminate the need for developing new vaccines or expanding access to existing ones.^6^

Post-mortem biological characterization of the specific causes of childhood deaths is unusual, especially in LMICs. Consequently, a significant portion of current meningitis estimates rely on imputation using non-specific tools such as verbal autopsy and vital registration data, which could lead to inaccurate estimation of specific meningitis etiologies and attribution of deaths to meningitis.^8^ Child Health and Mortality Prevention Surveillance (CHAMPS) can assist in recognizing the burden of meningitis deaths and specific etiologies through post-mortem minimally invasive tissue sampling (MITS), a procedure that uses biopsy needles to sample key organs and routine blood and cerebrospinal fluid (CSF) collection methods to enable post-mortem diagnosis. MITS is culturally appropriate and less resource-intensive for comprehensive disease and mortality surveillance than full autopsy.^9^, ^10^ This study aims to provide a detailed characterization of the specific pathogens and associated comorbidities responsible for meningitis-attributed deaths among under-5 children enrolled in the CHAMPS network.

## Methods

### Study design, setting, and data sources

Details of CHAMPS study methods have been published elsewhere.^11^, ^12^, ^13^ The study was conducted within defined catchment areas in six sub-Saharan Africa countries (Ethiopia, Kenya, Mali, Mozambique, Sierra Leone, and South Africa) and one South Asian country (Bangladesh). Site characteristics and inclusion criteria for MITS have been previously reported^11^ and are summarized in Fig. S1. Briefly, CHAMPS teams conduct active surveillance for stillbirths and deaths in children under 5. All deaths identified among catchment-area residents, whether occurring in the community or healthcare facility, were eligible for enrollment, with the goal to enroll and collect specimens within 24 h of death. The MITS timeframe may be extended up to 72 h after death if the decedent’s body was refrigerated. After obtaining parental consent, MITS procedures were conducted, including standardized methods to collect tissue specimens from multiple sites in the lung, liver, and brain, collection of body fluids (blood and CSF), and nasopharyngeal and rectal swabs.^14^, ^15^ Blood and CSF were cultured for bacteria. Blood was also screened for HIV, malaria and *Mycobacterium tuberculosis* using a nucleic acid amplification test, smears and rapid antigen tests, and GeneXpert, respectively. Brain, lung and liver tissue specimens were examined using routine histopathology, special stains, and immunohistochemistry as indicated.^16^ Multiplexed Taqman Array Cards were used to test for 126 organism targets, including 47 on CSF, using Real-Time polymerase chain reaction (PCR, Table S1); details of organism targets included in CHAMPS Taqman Array Cards have been previously reported.^17^ Ante-mortem clinical information (including whether a lumbar puncture was performed) was abstracted following standardized forms from medical records by CHAMPS-trained medical staff, and teams also conducted verbal autopsies through interviews with families.^18^

All information for each death was reviewed by a Determination of Cause of Death (DeCoDe) panel in each country.^18^ The DeCoDe panels are composed of specialists in pediatrics, obstetrics, infectious diseases, epidemiology and public health, histopathology and microbiology. Cause of death attribution followed the framework provided by the World Health Organization’s International Classification of Diseases, 10th Revision (ICD-10), and the application of ICD-10 to deaths during the perinatal period (ICD-PM).^19^ This framework incorporates the reporting of the underlying, antecedent (also known as intermediate), and immediate causes as part of the causal pathway to death.

DeCoDe panels attributed deaths to meningitis and identified meningitis etiologies based on CHAMPS-developed diagnosis standards^12^ (https://champshealth.org/wp-content/uploads/2021/01/CHAMPS-Diagnosis-Standards.pdf). To strengthen the specificity of the diagnosis, a level assignment system was used. Level 1 was assigned to meningitis deaths with histopathology showing pyogenic meningitis in brain tissue, together with documented meningitis symptoms. Level 1 was also assigned to specific etiologies when the pathogen was detected in brain tissue or CSF using special stains and immunohistochemistry.^16^ In cases where there was less specific evidence supporting a meningitis-attributed death, DeCoDe panels assigned level 2 (moderate evidence) or level 3 (weakest evidence) to the diagnosis, ensuring a graded approach to certainty based on available data (Table S2).

### Statistical analysis

For this report, we analyzed all cases in which meningitis was considered by the DeCoDe panel to have contributed to the causal pathway of death and in some analyses, included deaths attributed to causes other than meningitis as a comparison group. We present analyses stratified by site and by age group at death; age groups included deaths in first 24 h, early neonatal (1–6 days), late neonatal (7–27 days), early infancy (28 days to <6 months), late infancy (6 to <12 months), and children (12 to 59 months). All pathogens adjudicated by the DeCoDe panel to have contributed to the pathogenesis of meningitis were included in the analysis; co-infection with multiple pathogens means that the sum of organisms exceeds the number of deaths. Descriptive statistics were done, providing medians with interquartile ranges (IQR) for continuous variables and proportions for categorical variables. For weight-for-height, weight-for-age and height-for-age z-scores, normal was defined as ≥−2 standard deviation (SD), moderate as −3 to <−2 SD, and severe as <−3 SD using WHO standards. To examine the role of bacterial pathogens that are often acquired in hospital settings for meningitis deaths attributed to bacterial etiologies, we examined the distribution of pathogens based on whether the deaths occurred in the community or within 72 h following hospital admission compared with deaths determined by a DeCoDe panel to be hospital-associated or that occurred 72 or more hours after admission. Cause-specific mortality fractions for meningitis from DeCoDe as well as verbal autopsy diagnoses (both inSilico and interVA methods) were also calculated.

#### Ethics statement

Ethics committees overseeing investigators at each site and at Emory University approved overall and site-specific protocols (Emory IRB#: 00091706). Protocols are available at https://champshealth.org/resources/protocols. Parents or legal guardians provided written informed consent for the deceased’s participation prior to any MITS procedure.

Role of the funding source:

The funder participated in discussions of study design and data collection. They did not participate in the study's conduct; the management, analysis, or interpretation of the data; preparation, review, or approval of the manuscript; or decision to submit the manuscript for publication.

## Results

**Fig. 1:**
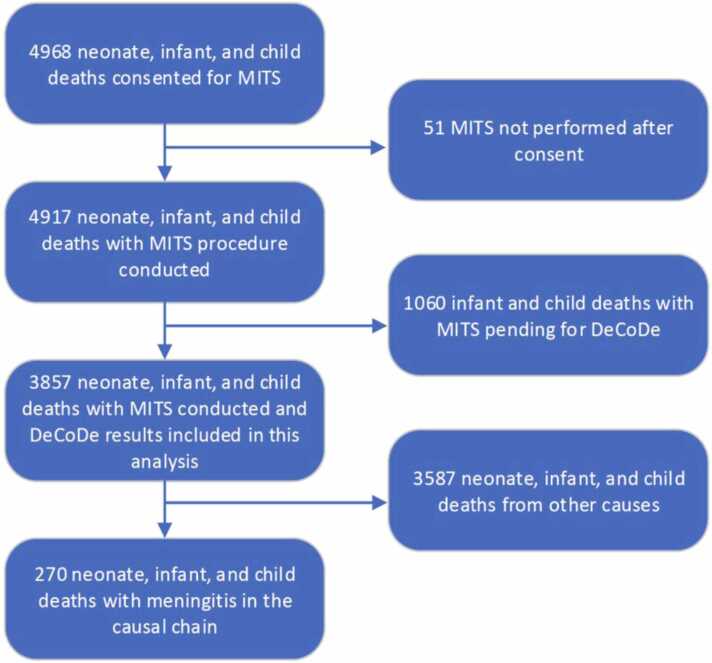
Flowchart of enrolled under-five deaths from CHAMPS sites between December 2016 – December 2023, that had minimally invasive tissue samples (MITS).

Between December 2016 and December 2023, CHAMPS teams identified a total of 10,277 deaths in under-5 children, and 79.6% (8189/10277) were enrolled in CHAMPS. Of those who were enrolled, 60.7% (4968/8189) consented for MITS, 98.9% (4917/4968) had MITS procedure conducted, and 77.6% (3857/4968) had DeCoDe completed by April 2024 (Fig. 1). Meningitis was identified as a contributing factor in the causal chain of death for 270 (7.0%) of the 3857 analyzed deaths (Fig. 1), of which 65.9% (178/270) were in neonates and 34.1% (92/270) were in infants or children (Table 1). The strength of evidence supporting meningitis as cause of death was provided for 261 deaths: with 81.2.% (212/261), 14.2% (37/270) and 4.5% (12/261) graded as Level 1, 2, and 3, respectively. Across sites, South Africa (16.6%, 150/904) and Ethiopia (15.6%, 52/333) had the highest proportion of under-5 children deaths attributed to meningitis, followed by Mali (6.7%, 18/269), and Sierra Leone (3.9%, 23/597). Conversely, meningitis was only implicated in 2.6% (9/351) of deaths in Bangladesh, 1.6% (13/818) in Mozambique and 0.9% (5/585) in Kenya (Table 1).

**Table 1:** Characteristics of under-five deaths with and without meningitis in the causal chain by age group, CHAMPS, December 2016 – December 2023.

	Neonates		Infants and Children	
	Meningitis -ve	Meningitis +ve	Meningitis -ve	Meningitis +ve
	N = 2219	N = 178	N = 1368	N = 92
**Age in days, median [IQR]**	1 [0, 4]	5 [3, 10]	349 [141, 714]	185 [64, 565]
**Male sex, (N,%)**	1263 (56.9)	112 (62.9)	745 (54.5)	62 (67.4)
**HIV status, (N,%)**				
Uninfected or unknown	1871 (84.3)	133 (74.7)	1101 (80.5)	66 (71.7)
HIV-exposed and uninfected	336 (15.1)	44 (24.7)	142 (10.4)	19 (20.7)
HIV-infected	12 ( 0.5)	1 (0.6)	125 ( 9.1)	7 (7.6)
**Weight at MITS (kg), median [IQR]**	2.0 [1.2, 2.9]	1.4 [1.0, 2.1]	6.6 [4.6, 9.3]	5.1 [3.1, 8.5]
**Weight-for-length Z-score, (N, %)** (N = 1438)				
Normal weight-for-length (>= −2SD)	–	–	589 (45.6)	34 (44.7)
Moderate wasting (<−2 SD, −3 SD)	–	–	204 (15.8)	11 (14.5)
Severe wasting (<−3 SD)	–	–	498 (38.6)	31 (40.8)
**Weight-for-age Z-score, (N, %)** (N = 1367)				
Normal weight-for-age (>= −2SD)	–	–	541 (40.2)	31 (34.1)
Moderate underweight (<−2 SD, −3 SD)	–	–	205 (15.2)	15 (16.5)
Severe underweight (< −3 SD)	–	–	601 (44.6)	45 (49.5)
**Length-for-age Z-score, (N, %)** (N = 1453)				
Normal length-for-age (>= −2SD)	–	–	790 (58.0)	43 (47.8)
Moderate stunning (<−2 SD, −3 SD)	–	–	193 (14.2)	11 (12.2)
Severe stunting (<- 3 SD)	–	–	380 (27.9)	36 (40.0)
**Birth weight, (N, %)**				
Extremely low birth weight (<1000 gm)	288 (13.0)	46 (25.8)	–	–
Very low birth weight (1000−1499 gm)	355 (16.0)	40 (22.5)	–	–
Low birth weight (1500−2499 gm)	470 (21.2)	33 (18.5)	–	–
Normal weight (2500−4000 gm)	693 (31.2)	27 (15.2)	–	–
Macrosomia (>4000 gm)	20 ( 0.9)	1 ( 0.6)	–	–
Missing	393 (17.7)	31 (17.4)	–	–
**Gestational age, (N, %)**				
<=28 weeks	344 (15.5)	48 (27.0)	23 ( 1.7)	8 ( 8.7)
28−33 weeks	304 (13.7)	32 (18.0)	42 ( 3.1)	5 ( 5.4)
34−36 weeks	222 (10.0)	12 ( 6.7)	66 ( 4.8)	9 ( 9.8)
37−42 weeks	604 (27.2)	23 (12.9)	210 (15.4)	6 ( 6.5)
Missing	743 (33.5)	63 (35.4)	1026 (75.1)	64 (69.6)
**Location of death, (N,%)**				
Community	130 ( 5.9)	11 ( 6.2)	398 (29.1)	18 (19.6)
Health facility	2089 (94.1)	167 (93.8)	970 (70.9)	74 (80.4)
**Hours between admission & death, median [IQR]** (N = 3046)	28 [8,72]	107 [47,162]	27 [9, 129]	177 [24, 930]
**Hours between death and MITS done, median [IQR]** (N = 3855)	9 [3, 18]	14 [5, 25]	13 [6, 21]	14 [8, 22]
**CHAMPS Sites, (N,%)**				
Bangladesh	333 (15.0)	8 ( 4.5)	9 ( 0.7)	1 ( 1.1)
Ethiopia	217 ( 9.8)	40 (22.5)	64 ( 4.7)	12 (13.0)
Kenya	245 (11.0)	1 ( 0.6)	335 (24.5)	4 ( 4.3)
Mali	166 ( 7.5)	8 ( 4.5)	85 ( 6.2)	10 (10.9)
Mozambique	557 (25.1)	5 ( 2.8)	248 (18.1)	8 ( 8.7)
Sierra Leone	238 (10.7)	11 ( 6.2)	336 (24.6)	12 (13.0)
South Africa	463 (20.9)	105 (59.0)	291 (21.3)	45 (48.9)

### Neonates

Among neonates (n = 178), 11.2% (20/178), 50% (89/178) ad 38.8%, (69/178) of meningitis-related deaths occurred within the first 24 h after birth, in early neonates (1–6 days of age) and among late neonates (7 to 28 days), respectively (Table 2). Among neonatal meningitis deaths, 62.9% (112/178) were males, 24.7% (44/178) were HIV-exposed, uninfected and one (0.6%) was in an HIV-infected baby. A large majority (80.9%, 119/147) of the neonatal meningitis deaths were in low birthweight (<2500 g) newborns (Table 1). Among 84.8% (151/178) meningitis-related neonatal deaths for which antemortem clinical records were available, 14.6% (22/151) had an antemortem diagnosis of meningitis and 22.5% (34/151) had a lumbar puncture done. The most common symptoms recorded antemortem for neonatal deaths from meningitis were seizures (25.2%, 38/151), loss of consciousness (20.5%, 31/151), fever (18.5%, 28/151), and altered mental status (18.5%, 28/151; Table S3).

**Table 2:** Causal chain classifications for under-five deaths with meningitis in the causal chain by age group, CHAMPS, December 2016 – December 2023.

	Total neonates	Death in first 24 h	Early neonate (1 to 6 days)	Late Neonate (7 to 27 days)	Total infants and children	Early infant (28 days - <6 months)	Late infant (6-<12 months)	Child (12-59 months)
	**N = 178**	N = 20	N = 89	N = 69	**N = 92**	N = 46	N = 16	N = 30
Meningitis as underlying	**6 (3.4)**	2 (10.0)	1 (1.1)	3 (4.3)	**7 (7.6)**	3 (6.5)	0 (0.0)	4 (13.3)
Meningitis as antecedent	**136 (76.4)**	13 (65.0)	74 (83.1)	49 (71.0)	**49 (53.3)**	26 (56.5)	7 (43.8)	16 (53.3)
Meningitis as immediate	**37 (20.8)**	5 (25.0)	15 (16.9)	17 (24.6)	**39 (42.4)**	18 (39.1)	9 (56.3)	12 (40.0)

The main comparison is between neonates and infants & children, in bold. We further stratified neonates into the following categories: death within the first 24 hours, early neonates, and late neonates. For infants & children, we further stratified them into late infants and children.

**Fig. 2:**
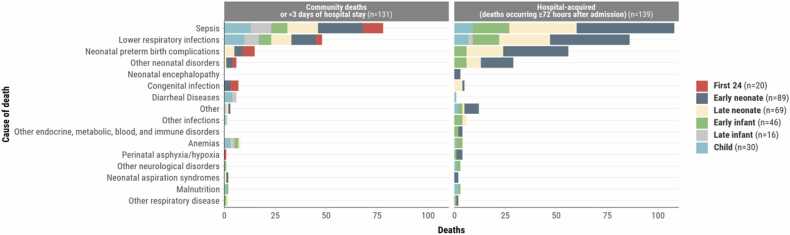
Most common comorbidities for meningitis deaths, by age group and by whether the meningitis death 1) occurred in the community or with fewer than 72 h in the hospital (N = 131 or 2) was determined to be hospital-associated or occurred 72 or more hours after hospital admission (N = 139), CHAMPS, December 2016 – December 2023.

Meningitis was often assigned as an antecedent/comorbid cause (76.4%, 136/178) among neonatal meningitis deaths; whilst it was an immediate cause in 20.8% ( 37/178) of cases and only infrequently an underlying cause (3.4%, 6/178; Table 2). The most frequent other conditions in the causal pathway observed among 178 neonatal deaths attributed to meningitis were sepsis (82.0%, 146/178), neonatal preterm birth complications (64.1%1, 14/178) and lower respiratory infections (51.7%, 92/178; Table 3, Fig. 2). The most common WHO ICD-PM underlying categories when meningitis was in the causal pathway were low birth weight/prematurity complications (52.8%, 94/178), infections (24.2%, 43/178), and complications of intrapartum events (10.1%, 18/178) (Table S5). Among those who had other infectious conditions in the causal pathway 89.9% (160/178), *Klebsiella pneumoniae* (56.9%, 91/160) and *Acinetobacter baumannii* (48.1%, 77/160) were common pathogens (Table S4). Among neonates who had both meningitis and sepsis in the causal pathway (n = 146), 131 (89.7%) had at least one of the same pathogens implicated as the cause of meningitis and sepsis. Among neonates who had both meningitis and lower respiratory infections in the causal pathway (n = 92), 78 (84.8%) had at least one of the same pathogens implicated in both the meningitis and the lower respiratory infections (Table S6a).

**Table 3:** Comorbidities for deaths attributed to meningitis, CHAMPS, December 2016 – December 2023.

	Total neonates	Death in first 24 h	Early neonate (1 to 6 days)	Late Neonate (7 to 27 days)	Total infants and children	Early infant (28 days - <6 months)	Late infant (6-<12 months)	Child (12-59 months)
	N = 178	N = 20	N = 89	N = 69	N = 92	N = 46	N = 16	N = 30
Sepsis	**146 (82.0)**	14 (70.0)	75 (84.3)	57 (82.6)	**68 (73.9)**	34 (73.9)	11 (68.8)	23 (76.7)
Lower respiratory infections	**92 (51.7)**	3 (15.0)	53 (59.6)	36 (52.2)	**56 (60.9)**	23 (50.0)	13 (81.2)	20 (66.7)
Neonatal preterm birth complications	**114 (64.0)**	9 (45.0)	64 (71.9)	41 (59.4)	**12 (13.0)**	12 (26.1)	0 (0)	0 (0)
Other neonatal disorders	**30 (16.9)**	2 (10.0)	19 (21.3)	9 (13.0)	**7 (7.6)**	7 (15.2)	0 (0)	0 (0)
Perinatal asphyxia/hypoxia	**21 (11.8)**	4 (20.0)	13 (14.6)	4 (5.8)	**2 (2.2)**	2 (4.3)	0 (0)	0 (0)
Congenital birth defects	**11 (6.2)**	1 (5.0)	2 (2.2)	8 (11.6)	**9 (9.8)**	7 (15.2)	1 (6.2)	1 (3.3)
Malnutrition	**1 (0.6)**	0 (0)	0 (0)	1 (1.4)	**19 (20.7)**	5 (10.9)	4 (25.0)	10 (33.3)
Congenital infection	**16 (9.0)**	6 (30.0)	5 (5.6)	5 (7.2)	**0 (0)**	0 (0)	0 (0)	0 (0)
Other	**10 (5.6)**	0 (0)	8 (9.0)	2 (2.9)	**5 (5.4)**	2 (4.3)	1 (6.2)	2 (6.7)
Anemias	**1 (0.6)**	0 (0)	0 (0)	1 (1.4)	**11 (12.0)**	5 (10.9)	2 (12.5)	4 (13.3)
Other infections	**4 (2.2)**	0 (0)	0 (0)	4 (5.8)	**6 (6.5)**	5 (10.9)	0 (0)	1 (3.3)
Diarrheal Diseases	**0 (0)**	0 (0)	0 (0)	0 (0)	**9 (9.8)**	1 (2.2)	2 (12.5)	6 (20.0)
HIV	**0 (0)**	0 (0)	0 (0)	0 (0)	**7 (7.6)**	2 (4.3)	2 (12.5)	3 (10.0)
Other endocrine, metabolic, blood, and immune disorders	**2 (1.1)**	0 (0)	2 (2.2)	0 (0)	**5 (5.4)**	3 (6.5)	2 (12.5)	0 (0)
Neonatal aspiration syndromes	**6 (3.4)**	0 (0)	5 (5.6)	1 (1.4)	**0 (0)**	0 (0)	0 (0)	0 (0)
Other neurological disorders	**0 (0)**	0 (0)	0 (0)	0 (0)	**6 (6.5)**	4 (8.7)	1 (6.2)	1 (3.3)
Injury	**0 (0)**	0 (0)	0 (0)	0 (0)	**5 (5.4)**	0 (0)	1 (6.2)	4 (13.3)
Other respiratory disease	**2 (1.1)**	0 (0)	1 (1.1)	1 (1.4)	**3 (3.3)**	2 (4.3)	0 (0)	1 (3.3)
Neonatal encephalopathy	**3 (1.7)**	0 (0)	3 (3.4)	0 (0)	**1 (1.1)**	1 (2.2)	0 (0)	0 (0)
Malaria	**0 (0)**	0 (0)	0 (0)	0 (0)	**3 (3.3)**	1 (2.2)	0 (0)	2 (6.7)
Kidney Disease	**0 (0)**	0 (0)	0 (0)	1 (1.4)	**1 (1.1)**	1 (2.2)	0 (0)	0 (0)
Other disorders of fluid, electrolyte and acid-base balance	**1 (0.6)**	0 (0)	1 (1.1)	0 (0)	**1 (1.1)**	1 (2.2)	0 (0)	0 (0)
Paralytic ileus and intestinal obstruction	**1 (0.6)**	0 (0)	1 (1.1)	0 (0)	**1 (1.1)**	0 (0)	0 (0)	1 (3.3)
Syphilis	**1 (0.6)**	0 (0)	1 (1.1)	0 (0)	**1 (1.1)**	1 (2.2)	0 (0)	0 (0)
Cancer	**0 (0)**	0 (0)	0 (0)	0 (0)	**1 (1.1)**	0 (0)	0 (0)	1 (3.3)
Chorioamnionitis and membrane complications	**1 (0.6)**	1 (5.0)	0 (0)	0 (0)	**0 (0)**	0 (0)	0 (0)	0 (0)
Liver disease	**0 (0)**	0 (0)	0 (0)	0 (0)	**1 (1.1)**	1 (2.2)	0 (0)	0 (0)
Other nutritional deficiencies	**0 (0)**	0 (0)	0 (0)	0 (0)	**1 (1.1)**	0 (0)	0 (0)	1 (3.3)
Poisoning	**0 (0)**	0 (0)	0 (0)	0 (0)	**1 (1.1)**	0 (0)	0 (0)	1 (3.3)
Tuberculosis	**0 (0)**	0 (0)	0 (0)	0 (0)	**1 (1.1)**	0 (0)	0 (0)	1 (3.3)
Upper respiratory infections	**0 (0)**	0 (0)	0 (0)	0 (0)	**1 (1.1)**	0 (0)	1 (6.2)	0 (0)
No other condition	7 (3.9)	1 (5.0)	1 (1.1)	5 (7.2)	3 (3.3)	0 (0)	0 (0)	3 (10.0)
>1 other condition	153 (86.0)	15 (75.0)	83 (93.3)	55 (79.7)	75 (81.5)	36 (78.3)	14 (87.5)	25 (83.3)

The main comparison is between neonates and infants & children, in bold. Neonates were further stratified into death within the first 24 hours, early neonates, and late neonates. For infants & children, the stratification was into late infants and children.

**Fig. 3:**
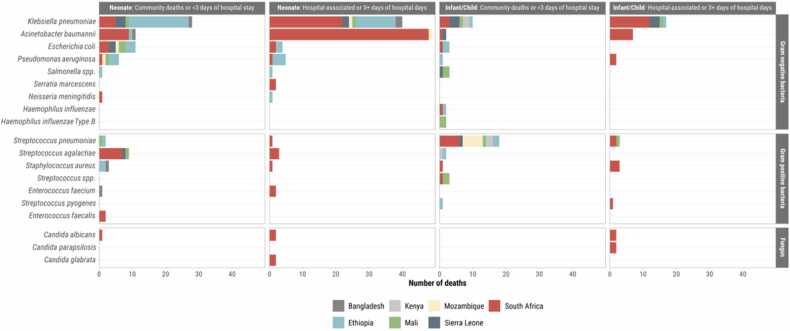
Pathogens attributed to meningitis deaths in children 1–59 months of age enrolled in CHAMPS, by CHAMPS site and by whether the death 1) occurred in the community or with fewer than 72 h in the hospital (N = 131) or 2) was determined to be hospital-associated or occurred 72 or more hours after hospital admission (N = 139), CHAMPS, December 2016 – December 2023. Pathogens implicated in at least three deaths were included.

Of the 178 meningitis-related neonatal deaths, 75.8% (135/178) were associated with at least one identified pathogen, 11.2% (20/178) had two pathogens, and 5.1% (9/178) had three implicated pathogens (Table 4, Table S7). No pathogen was detected in 7.9% (14/178). Among meningitis-related neonatal deaths. Fifty-six percent (55.6%, 99/178) of neonatal meningitis cases were hospital-associated, whereas 44.4% (79/178) were community-associated. Common pathogens for community-associated meningitis deaths were *K. pneumoniae* (35.4%, 28/79) and *Escherichia coli* (13.9%, 11/79). A third (35%, 7/20) of community-associated meningitis deaths in the first 24 hours after birth were due to *Streptococcus agalactiae (*Table 4, Table S7, S8, S9). Among 79 neonates with community-associated meningitis, 63 (79.7%) were also diagnosed with congenital/perinatal infection and/or sepsis, of which 32 (50.8%) were attributed to *K. pneumoniae* and 11 (17.5%) to *A. baumannii* (Table S10). Among hospital-associated meningitis-related neonatal deaths, common pathogens involved included *A. baumannii* (49.5%, 49/99) and *K. pneumoniae* (40.4%, 40/99; Table 4, Table S7, S8, S9). Among 99 neonates with hospital-associated meningitis, 87 (87.9%) were also diagnosed with congenital/perinatal infection or sepsis, of which 54 (62.1%) were attributed to *A. baumannii* and 48 (55.2%) to *K. pneumoniae*. The most frequent co-infections attributed to meningitis-related deaths among neonates were *K. pneumoniae* with *A. baumannii* (6.2%, 11/178), *P. aeruginosa* (3.4%, 6/178), or *E. coli* (3.4%, 6/178; Fig. S2).

**Table 4:** Pathogen profile for community- and hospital-associated meningitis by age group, CHAMPS, December 2016 – December 2023.

		Neonates		Infants and children	
Pathogens	All meningitis	Community deaths or <72 h of hospital stay	Hospital-associated or ≥72 h of hospital days	Community deaths or <72 h of hospital stay	Hospital-associated or ≥72 h of hospital days
	N = 270	N = 79	N = 99	N = 52	N = 40
**Gram negative bacteria**	**186 (68.9)**	**55 (69.6)**	**86 (86.9)**	**21 (40.4)**	**24 (60.0)**
*Klebsiella pneumoniae*	95 (35.2)	28 (35.4)	40 (40.4)	10 (19.2)	17 (42.5)
*Acinetobacter baumannii*	69 (25.6)	11 (13.9)	49 (49.5)	2 (3.8)	7 (17.5)
*Escherichia coli*	18 (6.7)	11 (13.9)	4 (4.0)	3 (5.8)	0 (0)
*Pseudomonas aeruginosa*	14 (5.2)	6 (7.6)	5 (5.1)	1 (1.9)	2 (5.0)
*Salmonella spp.*	5 (1.9)	1 (1.3)	1 (1.0)	3 (5.8)	0 (0)
*Neisseria meningitidis*	3 (1.1)	1 (1.3)	1 (1.0)	1 (1.9)	0 (0)
*Escherichia coli/Shigella spp.*	2 (0.7)	1 (1.3)	0 (0)	0 (0)	1 (2.5)
*NontypeableHaemophilus influenzae*	2 (0.7)	0 (0)	0 (0)	2 (3.8)	0 (0)
*Haemophilus influenzae Type A*	2 (0.7)	1 (1.3)	0 (0)	1 (1.9)	0 (0)
*Haemophilus influenzae Type B*	2 (0.7)	0 (0)	0 (0)	2 (3.8)	0 (0)
*Serratia marcescens*	2 (0.7)	0 (0)	2 (2.0)	0 (0)	0 (0)
*Citrobacter freundii*	1 (0.4)	1 (1.3)	0 (0)	0 (0)	0 (0)
*Enterobacter cloacae*	1 (0.4)	0 (0)	1 (1.0)	0 (0)	0 (0)
*Klebsiella oxytoca*	1 (0.4)	0 (0)	1 (1.0)	0 (0)	0 (0)
*Klebsiella spp.*	1 (0.4)	1 (1.3)	0 (0)	0 (0)	0 (0)
*Morganella morganii*	1 (0.4)	0 (0)	0 (0)	0 (0)	1 (2.5)
*Orientia tsutsugamushi*	1 (0.4)	1 (1.3)	0 (0)	0 (0)	0 (0)
*Proteus mirabilis*	1 (0.4)	1 (1.3)	0 (0)	0 (0)	0 (0)
*Vibrio cholerae*	1 (0.4)	1 (1.3)	0 (0)	0 (0)	0 (0)
**Gram positive bacteria**	**60 (22.2)**	**18 (22.8)**	**8 (8.1)**	**25 (48.1)**	**9 (22.5)**
*Streptococcus pneumoniae*	24 (8.9)	2 (2.5)	1 (1.0)	18 (34.6)	3 (7.5)
*Streptococcus agalactiae*	14 (5.2)	9 (11.4)	3 (3.0)	2 (3.8)	0 (0)
*Staphylococcus aureus*	8 (3.0)	3 (3.8)	1 (1.0)	1 (1.9)	3 (7.5)
*Streptococcus spp.*	4 (1.5)	0 (0)	1 (1.0)	3 (5.8)	0 (0)
*Enterococcus faecalis*	3 (1.1)	2 (2.5)	0 (0)	0 (0)	1 (2.5)
*Enterococcus faecium*	3 (1.1)	1 (1.3)	2 (2.0)	0 (0)	0 (0)
*Streptococcus pyogenes*	2 (0.7)	0 (0)	0 (0)	1 (1.9)	1 (2.5)
*Micrococcus species*	1 (0.4)	0 (0)	1 (1.0)	0 (0)	0 (0)
*Staphylococcus haemolyticus*	1 (0.4)	0 (0)	0 (0)	0 (0)	1 (2.5)
*Stenotrophomonas maltophilia*	1 (0.4)	1 (1.3)	0 (0)	0 (0)	0 (0)
**Viruses**	**3 (1.1)**	**1 (1.3)**	**0 (0)**	**1 (1.9)**	**1 (2.5)**
Adenovirus	1 (0.4)	0 (0)	0 (0)	0 (0)	1 (2.5)
Cytomegalovirus	1 (0.4)	0 (0)	0 (0)	1 (1.9)	0 (0)
Parechovirus	1 (0.4)	1 (1.3)	0 (0)	0 (0)	0 (0)
**Fungi**	**9 (3.3)**	**1 (1.3)**	**4 (4.0)**	**0 (0)**	**4 (10.0)**
*Candida albicans*	5 (1.9)	1 (1.3)	2 (2.0)	0 (0)	2 (5.0)
*Candida glabrata*	2 (0.7)	0 (0)	2 (2.0)	0 (0)	0 (0)
*Candida parapsilosis*	2 (0.7)	0 (0)	0 (0)	0 (0)	2 (5.0)
**Parasites**	**1 (0.4)**	**0 (0)**	**0 (0)**	**0 (0)**	**1 (2.5)**
*Toxoplasma gondii*	1 (0.4)	0 (0)	0 (0)	0 (0)	1 (2.5)
**Polymicrobial**	**45 (16.7)**	**11 (13.9)**	**18 (18.2)**	**8 (15.4)**	**8 (20.0)**
Number of cases with only one pathogen implicated	196 (72.6)	60 (75.9)	75 (75.8)	34 (65.4)	27 (67.5)
Number of cases with 2 pathogens implicated	35 (13.0)	8 (10.1)	12 (12.1)	7 (13.5)	8 (20.0)
Number of cases with 3 pathogens implicated	10 (3.7)	3 (3.8)	6 (6.1)	1 (1.9)	0 (0)
No pathogen detected	29 (10.7)	8 (10.1)	6 (6.1)	10 (19.2)	5 (12.5)

### Infants and children

Among 92 infant and child deaths related to meningitis, 50.0% (46/92) were among 28 days to <6-month-old infants, 67.4% (62/92) were male, 20.7% (19/92) were HIV exposed but uninfected and 7.6% (7/92) were in HIV infected infants or children. Regarding nutritional status, 49.5% (45/92), 40.0% (36/92), and 40.8% (31/92) deaths occurred in children who were severely underweight, stunted, and wasted, respectively, according to post-mortem measurements (Table 1). Antemortem clinical records were available for 82.6% (76/92); of these, 23.7% (18/76) had an antemortem diagnosis of meningitis and 15.8% (12/76) had a lumbar puncture done. The most common symptoms recorded antemortem were fever (52.6%, 40/76), vomiting (50.0%, 38/76), seizures (43.4%, 33/76), and altered mental status (40.8%, 31/76; Table S3).

Meningitis was typically determined to be the immediate (42.4%. 39/92) or antecedent/comorbid cause (53.3%, 49/92) and seldom the underlying cause (7.6%, 7/92) of infant and child deaths (Table 2). The most frequent other conditions in causal pathways observed among 92 infant and child deaths attributed to meningitis were sepsis (73.9%, 68/92), lower respiratory infections (60.9%, 56/92) and malnutrition (20.7%, 19/92; Table 3). Among infant and child deaths who had other infectious conditions in the causal pathway 88.0% (81/92), *K pneumoniae* (51.9%, 42/81) and *Streptococcus pneumoniae* (35.8%, 29/81) were common pathogens (Table S4). Among infants/children who had both meningitis and sepsis in the causal pathway (n = 68), a large majority (55, 80.9%) had at least one of the same pathogens implicated in both the meningitis and the sepsis. Likewise, those who had both meningitis and lower respiratory infections in the causal pathway (n = 56), 42 (75.0%) had at least one of the same pathogens implicated in both the meningitis and the lower respiratory infections (Table S6b).

Of the 92 meningitis-related infant and child deaths, 66.3% (61/92) were associated with one identified pathogen, 16.3% (15/92) with two pathogens and 1.1% (1/92) had three implicated pathogens; no pathogens were detected in 16.3% (15/92; Table 4, Table S7). Among meningitis-related infant and child deaths, just over half (56.5%, 52/92) were community-associated. Common pathogens for community-associated meningitis were *S. pneumoniae* (34.6%; 18/52) and *K. pneumoniae* (19.2%; 10/52; Table 4, S7, S9). Among 52 infant and child deaths with community-associated meningitis, 37 (71.2%) were also diagnosed with sepsis, of which the most common pathogens were *S. pneumoniae* (37.8%; 14/37) and *K. pneumoniae* (32.4%; 12/37) (Table S10). Among hospital-associated meningitis infant and child deaths, common pathogens were *K. pneumoniae* (42.5%, 17/40) and *A. baumannii* (17.5%, 7/40; Table 4, S6, S7). Among 40 infant and child deaths with hospital-associated meningitis, 31 (77.5%) were also diagnosed with sepsis, of which 23 (74.2%) were attributed to *K. pneumoniae* and 13 (41.9%) were attributed to *A. baumannii* (Table S10). The most frequent co-infections attributed to meningitis-related deaths among infants and children were *K. pneumoniae* with *A baumannii* (n = 4; Fig. S2).

### Both age groups

For community-associated meningitis, *K. pneumoniae* was most frequently detected in Ethiopia (51.4%, 19/37 of cases in Ethiopia), Sierra Leone (35.3%, 6/17), and South Africa (19.0%, 8/42), whereas *S. pneumoniae* was more common in Mozambique (54.5%, 6/11). For hospital-associated meningitis, *K. pneumoniae* was most prevalent in Ethiopia (86.9%, 13/15 of cases in Ethiopia) and South Africa (31.5%, 34/108). *A. baumannii* and *S. agalactiae* were predominantly found in South Africa, accounting for 23.8% (10/22) and 16.7% (7/22) of community-associated cases and 50.9% (55/108) and 2.8% (3/108) of hospital-associated cases, respectively (Table S11).

Among all 270 meningitis deaths, DeCoDe panels attributed meningitis as a cause of death based on multiple different results, including: postmortem culture (86.2%, 224/270), postmortem molecular diagnostics (TAC) (85.8%, 223/270), clinical pathological laboratory findings (81.5%, 212/270), immunohistochemistry (120/270, 46.2%) and others shown in Table S12. However, it is important to note that these procedures were considered essential for determining all causes of death for each child, which may include causes other than meningitis.

DeCoDe panel members deemed 84.8% (229/270) of meningitis deaths as potentially preventable (Table S13). Recommendations for how the deaths could have been prevented were provided for 96.1% (220/229) of preventable deaths and included improved infection prevention (67.3%), clinical management and quality of care (44.5%), antenatal care and obstetric care and management (27.3%) and changes in health-seeking behavior (26.8%; Table S14).

In the cohort of 3857 neonatal, infant, and child deaths, the proportion attributed to meningitis was higher from DeCoDe than from inSilico or interVA interpretations of verbal autopsy in Ethiopia (DeCoDe: 15.6%, inSilico: 8.7%, interVA: 8.4%) and South Africa (DeCoDe: 16.6%, inSilico: 1.1%, interVA: 0.8%), but lower in Kenya (DeCoDe: 0.9%, inSilico: 14.2%, interVA: 10.9%), Mozambique (DeCoDe: 1.6%, inSilico: 13.1%, interVA: 10.5%), and Sierra Leone (DeCoDe: 3.9%, inSilico: 14.1%, interVA: 9.5%) (Fig. S3). In Bangladesh and Mali, the proportions were similar across all methods. Among 270 DeCoDe-determined meningitis deaths, only 15 (5.6%) were correctly identified as meningitis by interVA and 20 (7.4%) by inSilico. Conversely, among 284 interVA and 360 inSilico, only 16 (5.6%) and 19 (5.3%), respectively, were confirmed by DeCoDe.

## Discussion

Our study revealed that meningitis contributed to 7.0% of under-5 deaths in the CHAMPS network, which is higher than the WHO’s estimation of 5.1%.^3^ This finding suggests that the underestimation of meningitis cases may result from not considering all the conditions in the causal chain. Additionally, it underscores the high prevalence of resistant organisms such as *K pneumoniae* and *A baumannii*. Consequently, without the administration of appropriate antibiotics alongside lumbar puncture, the under-five population faces a significant risk of mortality from meningitis. Although childhood vaccination has decreased the incidence of *S. pneumoniae* and Hib meningitis, S. pneumoniae remains a leading pathogen for community-acquired meningitis. Therefore, it is advisable to consider alternative antibiotic therapies, especially for low birth weight infants or children who have been hospitalized for several days.^20^, ^21^

Notable variations in under-5 deaths attributed to meningitis were observed across CHAMPS network sites, ranging from a very low proportion in Bangladesh to very high ones in Ethiopia or South Africa. This pattern is consistent with previous studies,^22^, ^23^ suggesting wide variability of regional disparities in meningitis-attributed mortality in sub-Saharan Africa and South Asia. Such disparities may be due to several factors, including variability in healthcare infrastructure, standard protocols, vaccination coverage, availability of public health interventions, health-seeking behavior, prevalent co-morbidities, socioeconomic factors, population densities, and environmental conditions.^24^, ^25^, ^26^ Additionally, as we are examining proportions rather than incidence rates, it is likely that the observed disparities are influenced by the higher prevalence of other diseases (e.g., malaria, pneumonia) in specific settings. Moreover, the inclusion of the neonatal population may contribute to a substantial number of very early deaths attributable to birth asphyxia at certain sites.

Our study demonstrated that deaths related to meningitis were more common among late neonates and young infants, which aligns with existing literature.^27^, ^28^, ^29^ The heightened susceptibility in late neonates and young infants may be attributed to their immature immune systems, absence of maternal antibodies if born premature/low birth weight and vulnerability to various pathogens.^30^ The primary mode of infection is often through bloodstream invasion, leading to central nervous system involvement, and in our study about two-thirds of meningitis deaths had concomitant sepsis. Early-onset infections primarily stem from maternal sources, as pregnancy and delivery expose the fetus or neonate to numerous pathogens, potentially transmitted through the vaginal canal or via contact with the neonate's skin during birth.^30^ Moreover, low birth weight is a recognized risk factor for neonatal meningitis, a factor present in over two-thirds of the neonates in our study. Conversely, late-onset infections typically originate in the healthcare setting, with medical devices such as endotracheal tubes, catheters, and feeding tubes posing an increased risk of infection.^31^, ^32^ Efforts to improve maternal and neonatal healthcare, develop specific maternal or childhood vaccines that can prevent meningitis in the first weeks of life, and increase awareness about early recognition and treatment of symptoms can play a significant role in reducing the burden of meningitis in young children.^33^ Among infants and children, undernutrition is an important co-factor explaining the poor outcome of meningitis in LMICs. Studies have shown that children who were underweight at the time of onset of meningitis have a substantially increased probability of neurological sequelae and death.^34^

More than half of meningitis cases were hospital-associated (more than half in neonates and just under half in infant and child age group), with a significant number of these cases being associated with sepsis. Previous studies have indicated that a significant portion of meningitis-related mortalities occur within the hospital setting are often sequelae to widespread sepsis.^35^, ^36^ Factors contributing to this include prolonged hospital stays, invasive medical procedures, and compromised immune systems, all of which elevate the likelihood of acquiring infections in healthcare facilities.^37^ Consequently, the high proportion of hospital-associated cases significantly influenced the pathogen profile observed in our study, particularly in locations like South Africa, where pathogens such as *A. baumannii*—a common hospital-acquired bacterium—were more prevalent. This contrasts with settings where community-acquired meningitis was more common, resulting in a different pathogen distribution. Enhancing infection prevention and control measures in healthcare settings is crucial for reducing the incidence of hospital-associated meningitis.^35^, ^36^

Community-associated meningitis deaths (47.4%) underscore the persistent burden of infections beyond healthcare settings, influenced by limited access to healthcare, delayed medical attention, and exposure to infectious agents.^38^, ^39^ Community-associated cases highlight the acuteness and life-threatening emergency that a meningitis case involves. Therefore, strategies may focus on enhancing community awareness, early recognition of symptoms, and improving access to healthcare services to facilitate prompt diagnosis and treatment.^38^, ^39^ Our study showed low accuracy of verbal autopsy for diagnosis of meningitis; therefore, we believe most meningitis deaths in the community remain miscategorized, especially among deaths that occur with little to no diagnostic evaluation.

Group B *Streptococcus* (GBS) is one of the leading causes of neonatal meningitis; despite significant advances in neonatal intensive care and recent efforts at prevention and early diagnosis of disease, mortality and morbidity remain high.^40^, ^41^ Adequate screening and prophylactic maternal antibiotics are not widely used and do not protect against late onset of GBS meningitis^30^, ^40^ but, if implemented, could save many lives. Our finding also suggests that the introduction of maternal vaccine to protect against GBS infection could have an important impact. Our study also highlighted the role of *K. pneumoniae* as an important cause of meningitis deaths among under 5, which has been reported in previous studies.^42^, ^43^, ^44^, ^45^, ^46^ However, asymptomatic maternal urinary tract infection during pregnancy and nosocomial bacterial colonization during the neonate’s hospital are noteworthy issues. Improving antenatal, intrapartum and delivery care, along with following rigorous protocol during NICU hospitalization, are essential to reduce the overall risk of nosocomial infections.^47^ Prevention strategies for maternal urinary tract infection could be integrated to enhance overall infant health outcomes. While our study also identified *A. baumannii* as an important cause at one of our sites (South Africa), we recognize the need for additional data to generalize this finding to the entire country or other countries.

The observed prevalence of *S. pneumoniae* among community-associated cases is in accord with this pathogen’s well-known role as leading causes of bacterial meningitis in children, particularly in low-resource settings.^40^, ^48^, ^49^ We hypothesize that the widespread use of vaccines has significantly reduced deaths from Hib, which was formerly the leading cause of meningitis in young children. Additionally, our study indicates a lower-than-expected proportion of deaths from *S pneumoniae* meningitis. However, *S. pneumoniae* remains one of the leading pathogens for community associated meningitis, suggesting potential gaps in coverage or vaccine type. The identification of fungal pathogens exclusively in hospital-associated cases, with *C. albicans* being the most common, raises concerns about the risk of nosocomial fungal infections in vulnerable populations, such as premature neonates and immunocompromised children. This finding highlights the importance of antifungal stewardship and infection control measures in healthcare settings.^50^, ^51^ Additionally, the rarity of viral and parasitic infections further emphasizes the dominant role of bacteria in meningitis deaths.

The fact that few meningitis-associated deaths had been recognized to have meningitis prior to death in our study population underscores the critical importance of timely identification and treatment in potentially reducing mortality rates. In addition, given the high proportion of deaths due to Klebsiella pneumoniae and Acinetobacter baumannii, it is imperative for health systems to implement culture techniques to identify these organisms and assess their sensitivities. Rapid diagnostic methods may also be necessary. Relying solely on lumbar puncture with cell count or turbidity assessment is insufficient for managing cases involving these concerning pathogens. Timely diagnosis allows healthcare providers to initiate appropriate therapies promptly, which can significantly improve outcomes for patients with meningitis.

There are limitations to the CHAMPS methodology, as previously described.^11^, ^12^, ^52^ These limitations include the overrepresentation of healthcare facility deaths, absence of traditional control groups in the CHAMPS data, direct comparisons between deceased and non-deceased children were not feasible. Furthermore, we grouped hospital-associated meningitis deaths with deaths occurring after more than 72 h of hospitalization, which might have included some cases where meningitis was initially acquired in the community. Conversely, some community-acquired or short-stay meningitis deaths may have had unrecognized exposure to the healthcare system. Additionally, regional differences in pathogen prevalence across sites, as seen in the high percentage of *Acinetobacter* and *Klebsiella* cases in South Africa, may skew the overall pathogen profiles observed. This is particularly relevant given that South Africa had a larger proportion of deaths compared to other sites. Moreover, meningitis can be a part of the wider sepsis syndrome, and some DeCoDe panels may have opted to label meningitis cases only as sepsis. Finally, we are unable to determine what proportion of pneumococcal meningitis deaths might have been vaccine preventable, as serotyping is not yet completed on these deaths.

In conclusion, our findings indicate that meningitis remains a leading cause of mortality among children under five, with many cases going undiagnosed before death, highlighting the urgent need for improved diagnostic and treatment methods. The high prevalence of pathogens such as *K pneumoniae* and *A baumannii*, coupled with the reduced incidence of *S pneumoniae* and Hib due to vaccination, underscores the need to develop vaccines targeting these emerging pathogens. Additionally, exploring alternative antibiotic therapies is crucial, especially for low-birth-weight infants or children with prolonged hospital stays. This emphasizes the importance of targeted interventions, including advanced diagnostic tools, effective treatment options, and well-equipped healthcare facilities. Strengthening surveillance and response strategies is essential for reducing meningitis-related mortality and improving child survival outcomes in these regions.

## Funding statement

This work was supported by the 10.13039/100000865Bill & Melinda Gates Foundation [OPP1126780] to Emory University.

## Disclaimer

Several authors are employed by the US Centers for Disease Control and Prevention (CDC). The findings and conclusions in this report are those of the authors and do not necessarily represent the official position of the CDC.

## Declaration of Competing Interest

The authors declare that they have no known competing financial interests or personal relationships that could have appeared to influence the work reported in this paper.
