# Supplementary material for: Etiologies and comorbidities of meningitis deaths in children under 5 years in high-mortality settings: Insights from the CHAMPS Network in the post-pneumococcal vaccine era
Source: J Infect. 2024 Dec;89(6):None. doi: 10.1016/j.jinf.2024.106341 (PMC11624489; doi:10.1016/j.jinf.2024.106341)
Supplement: Supplementary file 1 — Supplemental text 1 [file mmc1.docx]

| **Supplemental Table 1. Pathogen Targets (N = 116) Validated for Use on Child Health and Mortality Prevention Surveillance TaqMan Array Cards** | |
| --- | --- |
| **Pathogen** | **Target** |
| **Bacteria:** |  |
| *Acinetobacter baumannii* | R, B2 |
| *Aeromonas spp* | E |
| *Bartonella spp* | B2 |
| *Bordetella parapertussis/B. bronchiseptica (pIS1001)* | R |
| *Bordetella pertussis/B. holmesii (IS481)* | R |
| *Brucella spp* | B2 |
| *Burkholderia pseudomallei* | R, B2 |
| *Campylobacter coli* | E |
| *Campylobacter jejuni* | E |
| *Chlamydia pneumoniae* | R |
| *Chlamydia trachomatis* | R |
| *Clostridioides difficile, nontoxigenic* | E |
| *C. difficile toxin A tcdA* | E |
| *C. difficile toxin B tcdB* | E |
| *Corynebacterium diphtheria* | R |
| *Corynebacterium pseudotuberculosis/C. ulcerans* | R |
| *C. diphtheriae/C. pseudotuberculosis/ C. ulcerans diphtheria toxin tox* | R |
| *Enterococcus faecalis* | B2, E |
| *Enterococcus faecium* | B2, E |
| *Enteroinvasive E. coli/Shigella spp ipaH* | E |
| *Enteropathogenic E. coli bfpA* | E |
| *Enteropathogenic E. coli eae* | E |
| *Enteroaggregative E. coli aatA* | E |
| *Enteroaggregative E. coli/Shigella spp ipaH* | E |
| *Enterotoxigenic E. coli heat-labile toxin eltA* | E |
| *Enterotoxigenic E. coli heat-stable toxin STh estA* | E |
| *Enterotoxigenic E. coli heat-stable toxin STp estA* | E |
| *Haemophilus influenzae* | R, B1 |
| *H. influenzae type ba* | R, B1 |
| *Klebsiella pneumoniae* | R, B1 |
| *Leptospira* | B2 |
| *Moraxella catarrhalis* | R, B2 |
| *Mycobacterium tuberculosis* | R, B2, E |
| *Neisseria gonorrhoeae* | B2 |
| *Neisseria meningitidis* | B1 |
| *Orientia tsutsugamushi* | B1 |
| *Rickettsia spp* | B1 |
| *Salmonella enterica Paratyphi A* | B1 |
| *S. enterica Typhi* | B1 |
| *S. enterica/bongori* | B1, E |
| *Shiga toxin stx1* | E |
| *Shiga toxin stx2* | E |
| *Staphylococcus aureus* | R, B1 |
| *Streptococcus agalactiae (GBS)* | R, B1 |
| *Streptococcus pneumoniae* | R, B1 |
| *Streptococcus pyogenes (GAS)* | R, B1 |
| *Streptococcus suis* | B2 |
| *Treponema pallidum* | B1 |
| *Ureaplasma urealyticum/parvum* | B2 |
| *Vibrio cholerae cholera toxin ctxA* | E |

|  |  |  |  |
| --- | --- | --- | --- |
|  | **Supplemental Table 2: CHAMPS diagnosis standards for meningitis cases** | | |
|  |  |  |  |
|  | **Bacterial Meningitis** | |  |
|  | **ICD-10 Codes: G00.9** | |  |
|  |  |  |  |
|  | ***Panelists will list the etiologic agent on the appropriate line of the Panel Case Report Form unless listed above. Panelists will use clinical judgment in considering cerebrospinal fluid (CSF) results when CSF is contaminated with blood.*** | | |
|  |  |  |  |
|  | Level 1 |  | One of the following laboratory findings *in the absence of laboratory evidence of a causal viral pathogen*: |
|  | ·                 Histological evidence of purulent meningitis (no clinical symptoms required) | | |
|  | ·                 Positive lumbar puncture, defined as one of the following (no clinical symptoms required) | | |
|  | o   Bacteria observed on Gram stain of CSF | | |
|  | o   If antemortem tap was performed: | | |
|  | o   >100 leukocytes/mm3 CSF in the absence of a traumatic tap with >80% neutrophils, if differential is available | | |
|  | o   Between 10-100 leukocytes/mm3 CSF in the absence of traumatic tap and >80% neutrophils AND either >100 protein or <40 glucose | | |
|  | o   Isolation of bacterial pathogen from CSF culture | | |
|  | ·                 Detection of bacterial pathogen by CSF PCR (TAC) **and temperature >38.0 or <36.0 AND TWO of the following clinical signs and symptoms documented in the medical record:** | | |
|  | o   Convulsions | |  |
|  | o   Altered consciousness or coma | | |
|  | o   Lethargy or irritability | | |
|  | o   Apnea |  |  |
|  | o   Bulging fontanelle | |  |
|  | o   Neck stiffness | |  |
|  | o   Severe headache | |  |
|  |  |  |  |
|  | **Level 2** |  | **One of the following:** |
|  | ·                 **No laboratory data available and temperature >38.0 or <36.0 AND TWO of the following clinical signs and symptoms documented in the medical record:** | | |
|  | o   Convulsions | |  |
|  | o   Altered consciousness or coma | | |
|  | o   Lethargy or irritability | | |
|  | o   Apnea |  |  |
|  | o   Bulging fontanelle | |  |
|  | o   Neck stiffness | |  |
|  | o   Severe headache | |  |
|  | ·                 **One of the laboratory findings outlined above AND sudden onset of fever AND severe headache, stiff neck, or convulsions as reported by verbal autopsy** | | |
|  |  |  |  |
|  | **Level 3** |  | **One of the following:** |
|  | ·                 **Clinical diagnosis of meningitis with no lumbar puncture performed and no meninges available for histologic evaluation.** | | |
|  | ·                 **Sudden onset of fever AND severe headache, stiff neck, or convulsions as reported by verbal autopsy with no lumbar puncture performed and no meninges available for histologic evaluation.** | | |
|  | ·                 **Laboratory evidence of meningitis without sufficient clinical data or verbal autopsy symptoms for Level 1 or Level 2 diagnosis.** | | |
|  |  |  |  |
|  | **(Murray 2011) (WHO 2003) (Vergagno 2016)** | | |
|  |  |  |  |
|  | **Bacterial Meningitis due to a specific pathogen** | | |
|  | **ICD-10 Codes: A17 (Tuberculosis of the nervous system), A39.0 (meningococcal meningitis), G00.0 (Haemophilus meningitis)*,* G00.1 (Pneumococcal meningitis), G00.2 (Streptococcal meningitis), G00.3 (Staphylococcal meningitis), G00.8 (other bacterial), G00.9 (unspecified bacterial), G01 (meningitis in bacterial diseases classified elsewhere),** | | |
|  |  |  |  |
|  | Level 1 |  | Diagnosis of Level 1 meningitis as above with one of the following: |
|  | ·                 Histological evidence of purulent meningitis AND Immunohistochemical (IHC) evidence of a specific bacterial pathogen in brain tissue | | |
|  | ·                 Positive lumbar puncture for a specific pathogen defined as: | | |
|  | o   Isolation and identification of a specific bacterial pathogen from CSF culture | | |
|  | o   Identification of a specific bacterial pathogen from CSF special stain (if available) | | |
|  |  |  |  |
|  | Level 2 |  | Diagnosis of Level 2 meningitis with clinical findings as described above and detection of a specific bacterial pathogen by PCR (TAC) in CSF or brain tissue |
|  |  |  |  |
|  | Level 3 |  | Diagnosis of Level 3 meningitis as above with detection of a specific pathogen by PCR (TAC) in the CSF or brain tissue but without clinical symptoms to support Level 1 or Level 2 diagnosis |
|  |  |  |  |
|  | **Viral Meningitis or Encephalitis** | | |
|  | **ICD-10 Codes: A83 (Mosquito-borne viral encephalitis), A84 (Tick-borne viral encephalitis), A85 (other viral encephalitis), A86 (unspecified viral encephalitis), A87.0 (Enteroviral meningitis), A87.1 (Adenoviral meningitis), A87.2 (Lymphocytic choriomeningitis), A87.8 (other viral meningitis), A87.9 (unspecified viral meningitis), G02.0 (meningitis in viral diseases classified elsewhere)** | | |
|  |  |  |  |
|  | ***Panelists will reference ICD-10 and use the most specific etiologic agent for each case. If a specific code for that etiology does not exist, list the etiologic agent on the appropriate line on the Panel Case Report Form.*** | | |
|  |  |  |  |
|  | Level 1 |  | One of the following laboratory findings: |
|  | ·                 Lumbar puncture consistent with viral etiology, defined as all of the following: | | |
|  | o   >10 leukocytes/ mm3 and >50% lymphocytes in the absence of a traumatic tap | | |
|  | o   No bacteria observed on Gram stain of CSF | | |
|  | o   No bacteria recovered from CSF culture (if performed) | | |
|  | o   Negative latex agglutination test of CSF (if performed) | | |
|  | ·                 Detection of a virus likely to cause encephalitis in CSF by PCR (TAC) | | |
|  | ·                 CSF PCR positive for organism likely to cause encephalitis | | |
|  |  | AND one of the following medically documented clinical signs: | |
|  | ·                 **Convulsions** | |  |
|  | ·                 **Headache** | |  |
|  | ·                 **Neck stiffness** | | |
|  | ·                 **Confusion or altered mental status** | | |
|  | ·                 **Vomiting** | |  |
|  | ·                 **Bulging fontanelle** | | |
|  | ·                 **Cranial nerve palsies** | | |
|  |  |  |  |
|  | Level 2 |  | One of the laboratory findings outlined above AND TWO or more of the clinical signs above (convulsions, headache, neck stiffness, confusion, or vomiting) as reported by verbal autopsy. |
|  |  |  |  |
|  | **Level 3** |  | **Laboratory data, medical records or verbal autopsy supporting a diagnosis of viral meningitis or encephalitis but not meeting the criteria for Level 1 or Level 2 above.** |
|  |  |  |  |
|  | (Murray 2011) (Vergagno 2016) | | |

| **Table S3. Antemortem symptoms in clinical records for under-five deaths from meningitis, CHAMPS, December 2016 – December 2023 (N = 227*)** | | | | | | | | |
| --- | --- | --- | --- | --- | --- | --- | --- | --- |
|  | **Total neonates** | Death in first 24 hours | Early neonate (1 to 6 days) | Late Neonate (7 to 27 days) | **Total infants and children** | Early infant (1-6 months) | Late infant (6-12 months) | Child (12-59 months) |
| Symptoms | **N = 151** | N = 18 | N = 79 | N = 54 | **N = 76** | N = 43 | N = 10 | N = 23 |
| Fever | **28 (18.5)** | 3 ( 16.7) | 11 ( 13.9) | 14 ( 25.9) | **40 (52.6)** | 16 (37.2) | 7 ( 70.0) | 17 ( 73.9) |
| Seizures | **28 (18.5)** | 0 ( 0.0) | 21 ( 26.6) | 17 ( 31.5) | **33 (43.4)** | 19 (44.2) | 3 ( 30.0) | 11 ( 47.8) |
| Vomiting | **26 (17.2)** | 0 ( 0.0) | 9 ( 11.4) | 17 ( 31.5) | **38 (50.0)** | 23 (53.5) | 5 ( 50.0) | 10 ( 43.5) |
| Altered mental status | **28 (18.5)** | 1 ( 5.6) | 14 ( 17.7) | 13 ( 24.1) | **31 (40.8)** | 14 (32.6) | 5 ( 50.0) | 12 ( 52.2) |
| Loss of consciousness | **31 (20.5)** | 4 ( 22.2) | 15 ( 19.0) | 12 ( 22.2) | **24 (31.6)** | 15 (34.9) | 2 ( 20.0) | 7 ( 30.4) |
| Headache | **0 ( 0.0)** | 0 ( 0.0) | 0 ( 0.0) | 0 ( 0.0) | **8 (10.5)** | 2 ( 4.7) | 1 ( 10.0) | 5 ( 21.7) |
| Nuchal rigidity | **0 ( 0.0)** | 0 ( 0.0) | 0 ( 0.0) | 0 ( 0.0) | **5 (6.6)** | 4 ( 9.3) | 0 ( 0.0) | 1 ( 4.3) |
| *Restricted to deaths with any antemortem clinical records | | | |  |  |  |  |  |

| **Table S4. All pathogens in the causal chain for meningitis deaths including those associated with other comorbidities by age group, CHAMPS, December 2016 – December 2023** | | | |
| --- | --- | --- | --- |
| **Pathogens** | All meningitis | Total neonates | Total infants and children |
|  | N = 270 | N = 178 | N = 92 |
| **Gram negative bacteria** | **223 (82.6)** | **157 (88.2)** | **66 (71.7)** |
| *Klebsiella pneumoniae* | 133 (49.3) | 91 (51.1) | 42 (45.7) |
| *Acinetobacter baumannii* | 95 (35.2) | 77 (43.3) | 18 (19.6) |
| *Escherichia coli* | 35 (13.0) | 20 (11.2) | 15 (16.3) |
| *Pseudomonas aeruginosa* | 21 (7.8) | 15 (8.4) | 6 (6.5) |
| *Salmonella spp.* | 8 (3.0) | 4 (2.2) | 4 (4.3) |
| *Haemophilus influenzae Type A* | 5 (1.9) | 1 (0.6) | 4 (4.3) |
| *Escherichia coli/Shigella spp.* | 4 (1.5) | 1 (0.6) | 3 (3.3) |
| *NontypeableHaemophilus influenzae* | 4 (1.5) | 0 (0) | 4 (4.3) |
| *Enterobacter cloacae* | 3 (1.1) | 3 (1.7) | 0 (0) |
| *Neisseria meningitidis* | 3 (1.1) | 2 (1.1) | 1 (1.1) |
| *Serratia marcescens* | 3 (1.1) | 3 (1.7) | 0 (0) |
| *Haemophilus influenzae Type B* | 2 (0.7) | 0 (0) | 2 (2.2) |
| *Klebsiella oxytoca* | 2 (0.7) | 2 (1.1) | 0 (0) |
| *Morganella morganii* | 2 (0.7) | 1 (0.6) | 1 (1.1) |
| *Proteus mirabilis* | 2 (0.7) | 2 (1.1) | 0 (0) |
| *Treponema pallidum* | 2 (0.7) | 1 (0.6) | 1 (1.1) |
| *Bordetella spp.* | 1 (0.4) | 1 (0.6) | 0 (0) |
| *Citrobacter freundii* | 1 (0.4) | 1 (0.6) | 0 (0) |
| *Klebsiella spp.* | 1 (0.4) | 1 (0.6) | 0 (0) |
| *Klebsiella terragina* | 1 (0.4) | 1 (0.6) | 0 (0) |
| *Moraxella catarrhalis* | 1 (0.4) | 0 (0) | 1 (1.1) |
| *Orientia tsutsugamushi* | 1 (0.4) | 1 (0.6) | 0 (0) |
| *Proteus vulgaris* | 1 (0.4) | 1 (0.6) | 0 (0) |
| *Serratia spp.* | 1 (0.4) | 0 (0) | 1 (1.1) |
| *Shigella spp.* | 1 (0.4) | 0 (0) | 1 (1.1) |
| *Ureaplasma spp.* | 1 (0.4) | 1 (0.6) | 0 (0) |
| *Vibrio cholerae* | 1 (0.4) | 1 (0.6) | 0 (0) |
| **Gram positive bacteria** | **95 (35.2)** | **45 (25.3)** | **50 (54.3)** |
| *Streptococcus pneumoniae* | 33 (12.2) | 4 (2.2) | 29 (31.5) |
| *Staphylococcus aureus* | 17 (6.3) | 8 (4.5) | 9 (9.8) |
| *Streptococcus agalactiae* | 17 (6.3) | 15 (8.4) | 2 (2.2) |
| *Enterococcus faecium* | 14 (5.2) | 10 (5.6) | 4 (4.3) |
| *Streptococcus spp.* | 11 (4.1) | 4 (2.2) | 7 (7.6) |
| *Enterococcus faecalis* | 8 (3.0) | 5 (2.8) | 3 (3.3) |
| *Streptococcus pyogenes* | 2 (0.7) | 0 (0) | 2 (2.2) |
| *Listeria monocytogenes* | 1 (0.4) | 1 (0.6) | 0 (0) |
| *Micrococcus species* | 1 (0.4) | 1 (0.6) | 0 (0) |
| *Mycobacterium tuberculosis* | 1 (0.4) | 0 (0) | 1 (1.1) |
| *Staphylococcus haemolyticus* | 1 (0.4) | 0 (0) | 1 (1.1) |
| *Stenotrophomonas maltophilia* | 1 (0.4) | 1 (0.6) | 0 (0) |
| *Viridans streptococcus* | 1 (0.4) | 0 (0) | 1 (1.1) |
| **Viruses** | **20 (7.4)** | **4 (2.2)** | **16 (17.4)** |
| Human Immunodeficiency Virus | 7 (2.6) | 0 (0) | 7 (7.6) |
| Cytomegalovirus | 6 (2.2) | 1 (0.6) | 5 (5.4) |
| Adenovirus | 2 (0.7) | 0 (0) | 2 (2.2) |
| Influenza A | 2 (0.7) | 0 (0) | 2 (2.2) |
| Parainfluenza virus type 3 | 2 (0.7) | 1 (0.6) | 1 (1.1) |
| Respiratory syncytial virus | 2 (0.7) | 0 (0) | 2 (2.2) |
| Rhinovirus | 2 (0.7) | 0 (0) | 2 (2.2) |
| Enterovirus | 1 (0.4) | 1 (0.6) | 0 (0) |
| Human coronavirus OC43 | 1 (0.4) | 0 (0) | 1 (1.1) |
| Parechovirus | 1 (0.4) | 1 (0.6) | 0 (0) |
| **Fungi** | **18 (6.7)** | **8 (4.5)** | **10 (10.9)** |
| *Candida albicans* | 6 (2.2) | 3 (1.7) | 3 (3.3) |
| *Candida parapsilosis* | 4 (1.5) | 1 (0.6) | 3 (3.3) |
| *Pneumocystis jirovecii* | 3 (1.1) | 0 (0) | 3 (3.3) |
| *Candida auris* | 2 (0.7) | 2 (1.1) | 0 (0) |
| *Candida glabrata* | 2 (0.7) | 2 (1.1) | 0 (0) |
| *Candida spp.* | 1 (0.4) | 0 (0) | 1 (1.1) |
| **Parasites** | **4 (1.5)** | **0 (0)** | **4 (4.3)** |
| *Plasmodium falciparum* | 3 (1.1) | 0 (0) | 3 (3.3) |
| *Toxoplasma gondii* | 1 (0.4) | 0 (0) | 1 (1.1) |

| **Table S5. Underlying causes of death for neonatal deaths that had meningitis in the causal chain by age group and WHO ICD 10 Perinatal Mortality (PM) category, CHAMPS, December 2016 – December 2023** | | | | |
| --- | --- | --- | --- | --- |
|  | Total | Death in first 24 hours | Early neonate | Late Neonate |
|  | N = 178 | N = 20 | N = 89 | N = 69 |
| Congenital malformations, deformations & chromosomal abnormalities N1 | 11 (6.2) | 1 (5.0) | 2 (2.2) | 8 (11.0) |
| Disorders related to fetal growth N2 | 1 (0.6) | 0 (0) | 0 (0) | 1 (1.4) |
| Birth trauma N3 | 0 (0) | 0 (0) | 0 (0) | 0 (0) |
| Complications of intrapartum events N4 | 18 (10.1) | 4 (20.0) | 10 (11.1) | 4 (5.5) |
| Convulsion and disorder of cerebral Status N5 | 0 (0) | 0 (0) | 0 (0) | 0 (0) |
| Infections N6 | 43 (24.2) | 9 (45.0) | 14 (15.6) | 20 (27.4) |
| Respiratory and cardiovascular disorders N7 | 9 (5.1) | 3 (15.0) | 6 (6.7) | 0 (0) |
| Other neonatal conditions N8 | 2 (1.1) | 0 (0) | 1 (1.1) | 1 (1.4) |
| Low birth weight/prematurity complications N9 | 94 (52.8) | 3 (15.0) | 56 (62.2) | 35 (47.9) |
| Miscellaneous N10 | 0 (0) | 0 (0) | 0 (0) | 0 (0) |
| Unspecified condition N11 | 0 (0) | 0 (0) | 0 (0) | 0 (0) |

| **Table S6a.** Pathogens attributed to meningitis and sepsis for 146 neonatal deaths with both meningitis and sepsis in the causal chain, and pathogens attributed to meningitis and lower respiratory infections for 92 neonatal deaths with both meningitis and lower respiratory infections in the causal chain. | | | | | | |
| --- | --- | --- | --- | --- | --- | --- |
|  | Meningitis and sepsis in causal chain (N = 146) | | | Meningitis and lower respiratory infections in causal chain (N = 92) | | |
| Pathogen | Implicated in both meningitis and sepsis | Implicated in meningitis but not sepsis | Implicated in sepsis but not meningitis | Implicated in both meningitis and lower respiratory infections | Implicated in meningitis but not lower respiratory infections | Implicated in lower respiratory infections but not meningitis |
| *Acinetobacter baumannii* | 51 (34.9) | 1 (0.7) | 12 (8.2) | 46 (50.0) | 2 (2.2) | 9 (9.8) |
| *Candida albicans* | 1 (0.7) | 0 (0) | 0 (0) | 1 (1.1) | 0 (0) | 0 (0) |
| *Candida auris* | 0 (0) | 0 (0) | 2 (1.4) | 0 (0) | 0 (0) | 0 (0) |
| *Candida glabrata* | 1 (0.7) | 0 (0) | 0 (0) | 1 (1.1) | 0 (0) | 0 (0) |
| *Citrobacter freundii* | 1 (0.7) | 0 (0) | 0 (0) | 0 (0) | 0 (0) | 0 (0) |
| *Enterobacter cloacae* | 0 (0) | 1 (0.7) | 2 (1.4) | 0 (0) | 0 (0) | 0 (0) |
| *Enterococcus faecalis* | 0 (0) | 2 (1.4) | 2 (1.4) | 0 (0) | 1 (1.1) | 2 (2.2) |
| *Enterococcus faecium* | 3 (2.1) | 0 (0) | 7 (4.8) | 0 (0) | 0 (0) | 0 (0) |
| Enterovirus | 0 (0) | 0 (0) | 1 (0.7) | 0 (0) | 0 (0) | 0 (0) |
| *Escherichia coli/Shigella spp.* | 9 (6.2) | 3 (2.1) | 3 (2.1) | 2 (2.2) | 6 (6.5) | 1 (1.1) |
| *Haemophilus influenzae* Type A | 1 (0.7) | 0 (0) | 0 (0) | 1 (1.1) | 0 (0) | 0 (0) |
| *Klebsiella oxytoca* | 1 (0.7) | 0 (0) | 1 (0.7) | 0 (0) | 1 (1.1) | 0 (0) |
| *Klebsiella pneumoniae* | 58 (39.7) | 3 (2.1) | 21 (14.4) | 34 (37.0) | 2 (2.2) | 8 (8.7) |
| *Klebsiella spp.* | 0 (0) | 1 (0.7) | 0 (0) | 0 (0) | 0 (0) | 0 (0) |
| *Klebsiella terragina* | 0 (0) | 0 (0) | 1 (0.7) | 0 (0) | 0 (0) | 0 (0) |
| *Micrococcus species* | 0 (0) | 1 (0.7) | 0 (0) | 0 (0) | 0 (0) | 0 (0) |
| *Morganella morganii* | 0 (0) | 0 (0) | 1 (0.7) | 0 (0) | 0 (0) | 0 (0) |
| *Neisseria meningitidis* | 2 (1.4) | 0 (0) | 0 (0) | 1 (1.1) | 1 (1.1) | 0 (0) |
| *Orientia tsutsugamushi* | 1 (0.7) | 0 (0) | 0 (0) | 1 (1.1) | 0 (0) | 0 (0) |
| *Proteus mirabilis* | 0 (0) | 0 (0) | 1 (0.7) | 0 (0) | 0 (0) | 0 (0) |
| *Proteus vulgaris* | 0 (0) | 0 (0) | 1 (0.7) | 0 (0) | 0 (0) | 0 (0) |
| *Pseudomonas aeruginosa* | 9 (6.2) | 1 (0.7) | 2 (1.4) | 4 (4.3) | 3 (3.3) | 2 (2.2) |
| *Salmonella spp.* | 1 (0.7) | 0 (0) | 2 (1.4) | 0 (0) | 1 (1.1) | 0 (0) |
| *Serratia marcescens* | 1 (0.7) | 0 (0) | 0 (0) | 0 (0) | 1 (1.1) | 1 (1.1) |
| *Staphylococcus aureus* | 2 (1.4) | 2 (1.4) | 3 (2.1) | 2 (2.2) | 1 (1.1) | 1 (1.1) |
| *Stenotrophomonas maltophilia* | 0 (0) | 1 (0.7) | 0 (0) | 0 (0) | 0 (0) | 0 (0) |
| *Streptococcus agalactiae* | 10 (6.8) | 0 (0) | 3 (2.1) | 2 (2.2) | 0 (0) | 0 (0) |
| *Streptococcus pneumoniae* | 1 (0.7) | 0 (0) | 1 (0.7) | 1 (1.1) | 1 (1.1) | 1 (1.1) |
| *Streptococcus spp.* | 0 (0) | 1 (0.7) | 1 (0.7) | 0 (0) | 0 (0) | 2 (2.2) |
| *Vibrio cholerae* | 1 (0.7) | 0 (0) | 0 (0) | 0 (0) | 1 (1.1) | 0 (0) |

| **Table S6b.** Pathogens attributed to meningitis and sepsis for 68 infant/child deaths with both meningitis and sepsis in the causal chain, and pathogens attributed to meningitis and lower respiratory infections for 56 infant/child deaths with both meningitis and lower respiratory infections in the causal chain. | | | | | | |
| --- | --- | --- | --- | --- | --- | --- |
|  | Meningitis and sepsis in causal chain (N = 68) | | | Meningitis and lower respiratory infections in causal chain (N = 56) | | |
| Pathogen | Implicated in both meningitis and sepsis | Implicated in meningitis but not sepsis | Implicated in sepsis but not meningitis | Implicated in both meningitis and lower respiratory infections | Implicated in meningitis but not lower respiratory infections | Implicated in lower respiratory infections but not meningitis |
| *Acinetobacter baumannii* | 6 (8.8) | 1 (1.5) | 8 (11.8) | 4 (7.1) | 1 (1.8) | 5 (8.9) |
| *Adenovirus* | 0 (0) | 1 (1.5) | 0 (0) | 1 (1.8) | 0 (0) | 0 (0) |
| *Candida albicans* | 2 (2.9) | 0 (0) | 1 (1.5) | 1 (1.8) | 0 (0) | 0 (0) |
| *Candida parapsilosis* | 1 (1.5) | 1 (1.5) | 1 (1.5) | 0 (0) | 2 (3.6) | 0 (0) |
| *Candida spp.* | 0 (0) | 0 (0) | 1 (1.5) | 0 (0) | 0 (0) | 0 (0) |
| *Cytomegalovirus* | 1 (1.5) | 0 (0) | 2 (2.9) | 1 (1.8) | 0 (0) | 3 (5.4) |
| *Enterococcus faecalis* | 1 (1.5) | 0 (0) | 1 (1.5) | 1 (1.8) | 0 (0) | 1 (1.8) |
| *Enterococcus faecium* | 0 (0) | 0 (0) | 3 (4.4) | 0 (0) | 0 (0) | 0 (0) |
| *Escherichia coli/Shigella spp.* | 3 (4.4) | 0 (0) | 10 (14.7) | 2 (3.6) | 1 (1.8) | 1 (1.8) |
| *Nontypeable Haemophilus influenzae* | 0 (0) | 1 (1.5) | 0 (0) | 2 (3.6) | 0 (0) | 2 (3.6) |
| *Haemophilus influenzae Type A* | 0 (0) | 0 (0) | 2 (2.9) | 1 (1.8) | 0 (0) | 3 (5.4) |
| *Haemophilus influenzae Type B* | 2 (2.9) | 0 (0) | 0 (0) | 1 (1.8) | 0 (0) | 0 (0) |
| *Klebsiella pneumoniae* | 21 (30.9) | 2 (2.9) | 14 (20.6) | 14 (25.0) | 4 (7.1) | 8 (14.3) |
| *Morganella morganii* | 1 (1.5) | 0 (0) | 0 (0) | 0 (0) | 0 (0) | 0 (0) |
| *Pneumocystis jirovecii* | 0 (0) | 0 (0) | 1 (1.5) | 0 (0) | 0 (0) | 0 (0) |
| *Pseudomonas aeruginosa* | 2 (2.9) | 1 (1.5) | 0 (0) | 2 (3.6) | 1 (1.8) | 2 (3.6) |
| *Salmonella spp.* | 3 (4.4) | 0 (0) | 0 (0) | 0 (0) | 1 (1.8) | 0 (0) |
| *Serratia spp.* | 0 (0) | 0 (0) | 1 (1.5) | 0 (0) | 0 (0) | 0 (0) |
| *Staphylococcus aureus* | 4 (5.9) | 0 (0) | 2 (2.9) | 3 (5.4) | 0 (0) | 4 (7.1) |
| *Streptococcus agalactiae* | 1 (1.5) | 1 (1.5) | 0 (0) | 0 (0) | 2 (3.6) | 0 (0) |
| *Streptococcus pneumoniae* | 13 (19.1) | 1 (1.5) | 5 (7.4) | 16 (28.6) | 0 (0) | 4 (7.1) |
| *Streptococcus pyogenes* | 1 (1.5) | 0 (0) | 0 (0) | 0 (0) | 0 (0) | 0 (0) |
| *Streptococcus spp.* | 2 (2.9) | 0 (0) | 2 (2.9) | 0 (0) | 1 (1.8) | 2 (3.6) |
| *Toxoplasma gondii* | 0 (0) | 1 (1.5) | 0 (0) | 0 (0) | 1 (1.8) | 0 (0) |

| **Table S7. Pathogens attributed to meningitis by age group, CHAMPS, December 2016 – December 2023** | | | | | | | | |
| --- | --- | --- | --- | --- | --- | --- | --- | --- |
| **Pathogens** | **Total neonates** | Death in first 24 hours | Early neonate (1 to 6 days) | Late Neonate (7 to 27 days) | **Total infants and children** | Early infant (28 days-<6 months) | Late infant (6-<12 months) | Child (12-59 months) |
|  | **N = 178** | N = 20 | N = 89 | N = 69 | **N = 92** | N = 46 | N = 16 | N = 30 |
| **Gram negative bacteria** | **141 (79.2)** | **9 (45.0)** | **80 (89.9)** | **52 (75.4)** | **45 (48.9)** | **27 (58.7)** | **4 (25.0)** | **14 (46.7)** |
| *Klebsiella pneumoniae* | **68 (38.2)** | 8 (40.0) | 43 (48.3) | 17 (24.6) | **27 (29.3)** | 18 (39.1) | 2 (12.5) | 7 (23.3) |
| *Acinetobacter baumannii* | **60 (33.7)** | 0 (0) | 34 (38.2) | 26 (37.7) | **9 (9.8)** | 6 (13.0) | 1 (6.2) | 2 (6.7) |
| *Escherichia coli* | **15 (8.4)** | 3 (15.0) | 8 (9.0) | 4 (5.8) | **3 (3.3)** | 2 (4.3) | 0 (0) | 1 (3.3) |
| *Pseudomonas aeruginosa* | **11 (6.2)** | 1 (5.0) | 6 (6.7) | 4 (5.8) | **3 (3.3)** | 1 (2.2) | 0 (0) | 2 (6.7) |
| *Salmonella spp.* | **2 (1.1)** | 0 (0) | 1 (1.1) | 1 (1.4) | **3 (3.3)** | 1 (2.2) | 0 (0) | 2 (6.7) |
| *Neisseria meningitidis* | **2 (1.1)** | 0 (0) | 1 (1.1) | 1 (1.4) | **1 (1.1)** | 0 (0) | 0 (0) | 1 (3.3) |
| *Escherichia coli/Shigella spp.* | **1 (0.6)** | 0 (0) | 0 (0) | 1 (1.4) | **1 (1.1)** | 0 (0) | 0 (0) | 1 (3.3) |
| *NontypeableHaemophilus influenzae* | **0 (0)** | 0 (0) | 0 (0) | 0 (0) | **2 (2.2)** | 0 (0) | 0 (0) | 2 (6.7) |
| *Haemophilus influenzae Type A* | **1 (0.6)** | 0 (0) | 1 (1.1) | 0 (0) | **1 (1.1)** | 1 (2.2) | 0 (0) | 0 (0) |
| *Haemophilus influenzae Type B* | **0 (0)** | 0 (0) | 0 (0) | 0 (0) | **2 (2.2)** | 1 (2.2) | 1 (6.2) | 0 (0) |
| *Serratia marcescens* | **2 (1.1)** | 0 (0) | 0 (0) | 2 (2.9) | **0 (0)** | 0 (0) | 0 (0) | 0 (0) |
| *Citrobacter freundii* | **1 (0.6)** | 0 (0) | 0 (0) | 1 (1.4) | **0 (0)** | 0 (0) | 0 (0) | 0 (0) |
| *Enterobacter cloacae* | **1 (0.6)** | 0 (0) | 1 (1.1) | 0 (0) | **0 (0)** | 0 (0) | 0 (0) | 0 (0) |
| *Klebsiella oxytoca* | **1 (0.6)** | 0 (0) | 0 (0) | 1 (1.4) | **0 (0)** | 0 (0) | 0 (0) | 0 (0) |
| *Klebsiella spp.* | **1 (0.6)** | 0 (0) | 1 (1.1) | 0 (0) | **0 (0)** | 0 (0) | 0 (0) | 0 (0) |
| *Morganella morganii* | **0 (0)** | 0 (0) | 0 (0) | 0 (0) | **1 (1.1)** | 0 (0) | 0 (0) | 1 (3.3) |
| *Orientia tsutsugamushi* | **1 (0.6)** | 0 (0) | 0 (0) | 1 (1.4) | **0 (0)** | 0 (0) | 0 (0) | 0 (0) |
| *Proteus mirabilis* | **1 (0.6)** | 0 (0) | 0 (0) | 1 (1.4) | **0 (0)** | 0 (0) | 0 (0) | 0 (0) |
| *Vibrio cholerae* | **1 (0.6)** | 0 (0) | 0 (0) | 1 (1.4) | **0 (0)** | 0 (0) | 0 (0) | 0 (0) |
| **Gram positive bacteria** | **26 (14.6)** | **9 (45.0)** | **3 (3.4)** | **14 (20.3)** | **34 (37.0)** | **14 (30.4)** | **9 (56.2)** | **11 (36.7)** |
| *Streptococcus pneumoniae* | **3 (1.7)** | 2 (10.0) | 1 (1.1) | 0 (0) | **21 (22.8)** | 7 (15.2) | 7 (43.8) | 7 (23.3) |
| *Streptococcus agalactiae* | **12 (6.7)** | 7 (35.0) | 0 (0) | 5 (7.2) | **2 (2.2)** | 0 (0) | 1 (6.2) | 1 (3.3) |
| *Staphylococcus aureus* | **4 (2.2)** | 0 (0) | 0 (0) | 4 (5.8) | **4 (4.3)** | 3 (6.5) | 0 (0) | 1 (3.3) |
| *Streptococcus spp.* | **1 (0.6)** | 0 (0) | 1 (1.1) | 0 (0) | **3 (3.3)** | 1 (2.2) | 1 (6.2) | 1 (3.3) |
| *Enterococcus faecalis* | **2 (1.1)** | 0 (0) | 0 (0) | 2 (2.9) | **1 (1.1)** | 0 (0) | 0 (0) | 1 (3.3) |
| *Enterococcus faecium* | **3 (1.7)** | 0 (0) | 0 (0) | 3 (4.3) | **0 (0)** | 0 (0) | 0 (0) | 0 (0) |
| *Streptococcus pyogenes* | **0 (0)** | 0 (0) | 0 (0) | 0 (0) | **2 (2.2)** | 2 (4.3) | 0 (0) | 0 (0) |
| *Micrococcus species* | **1 (0.6)** | 0 (0) | 1 (1.1) | 0 (0) | **0 (0)** | 0 (0) | 0 (0) | 0 (0) |
| *Staphylococcus haemolyticus* | **0 (0)** | 0 (0) | 0 (0) | 0 (0) | **1 (1.1)** | 1 (2.2) | 0 (0) | 0 (0) |
| *Stenotrophomonas maltophilia* | **1 (0.6)** | 0 (0) | 1 (1.1) | 0 (0) | **0 (0)** | 0 (0) | 0 (0) | 0 (0) |
| **Virus** | **1 (0.6)** | **0 (0)** | **0 (0)** | **1 (1.4)** | **2 (2.2)** | **1 (2.2)** | **1 (6.2)** | **0 (0)** |
| Adenovirus | **0 (0)** | 0 (0) | 0 (0) | 0 (0) | **1 (1.1)** | 0 (0) | 1 (6.2) | 0 (0) |
| Cytomegalovirus | **0 (0)** | 0 (0) | 0 (0) | 0 (0) | **1 (1.1)** | 1 (2.2) | 0 (0) | 0 (0) |
| Parechovirus | **1 (0.6)** | 0 (0) | 0 (0) | 1 (1.4) | **0 (0)** | 0 (0) | 0 (0) | 0 (0) |
| **Fungus** | **5 (2.8)** | **0 (0)** | **0 (0)** | **5 (7.2)** | **4 (4.3)** | **4 (8.7)** | **0 (0)** | **0 (0)** |
| *Candida albicans* | **3 (1.7)** | 0 (0) | 0 (0) | 3 (4.3) | **2 (2.2)** | 2 (4.3) | 0 (0) | 0 (0) |
| *Candida glabrata* | **2 (1.1)** | 0 (0) | 0 (0) | 2 (2.9) | **0 (0)** | 0 (0) | 0 (0) | 0 (0) |
| *Candida parapsilosis* | **0 (0)** | 0 (0) | 0 (0) | 0 (0) | **2 (2.2)** | 2 (4.3) | 0 (0) | 0 (0) |
| **Parasite** | **0 (0)** | **0 (0)** | **0 (0)** | **0 (0)** | **1 (1.1)** | **0 (0)** | **0 (0)** | **1 (3.3)** |
| *Toxoplasma gondii* | **0 (0)** | 0 (0) | 0 (0) | 0 (0) | **1 (1.1)** | 0 (0) | 0 (0) | 1 (3.3) |
| **Polymicrobial** | **29 (16.3)** | **3 (15.0)** | **14 (15.7)** | **12 (17.4)** | **16 (17.4)** | **8 (17.4)** | **2 (12.5)** | **6 (20.0)** |
| **Number of cases with only one pathogen implicated** | **135 (75.8)** | **15 (75.0)** | **68 (76.4)** | **52 (75.4)** | **61 (66.3)** | **33 (71.7)** | **10 (62.5)** | **18 (60.0)** |
| **Number of cases with 2 pathogens implicated** | **20 (11.2)** | **3 (15.0)** | **10 (11.2)** | **7 (10.1)** | **15 (16.3)** | **8 (17.4)** | **2 (12.5)** | **5 (16.7)** |
| **Number of cases with 3 pathogens implicated** | **9 (5.1)** | **0 (0)** | **4 (4.5)** | **5 (7.2)** | **1 (1.1)** | **0 (0)** | **0 (0)** | **1 (3.3)** |
| **No pathogen detected** | **14 (7.9)** | **2 (10.0)** | **7 (7.9)** | **5 (7.2)** | **15 (16.3)** | **5 (10.9)** | **4 (25.0)** | **6 (20.0)** |

| **Table S8. Pathogen profile by age group for hospital-associated meningitis, CHAMPS, December 2016 – December 2023** | | | | | | | | |
| --- | --- | --- | --- | --- | --- | --- | --- | --- |
| **Pathogens** | **Total neonates** | Death in first 24 hours | Early neonate (1 to 6 days) | Late Neonate (7 to 27 days) | **Total infants and children** | Early infant (28 days-<6 months) | Late infant (6-<12 months) | Child (12-59 months) |
|  | N = 238 | N = 139 | N = 61 | N = 38 | N = 40 | N = 28 | N = 2 | N = 10 |
| **Gram negative bacteria** | 196 (82.4) | **110 (79.1)** | **56 (91.8)** | **30 (78.9)** | 24 (60) | **16 (57.1)** | **1 (50.0)** | **7 (70.0)** |
| *Klebsiella pneumoniae* | 97 (40.8) | 57 (41.0) | 31 (50.8) | 9 (23.7) | 17 (42.5) | 13 (46.4) | 0 (0) | 4 (40.0) |
| *Acinetobacter baumannii* | 105 (44.1) | 56 (40.3) | 29 (47.5) | 20 (52.6) | 7 (17.5) | 4 (14.3) | 1 (50.0) | 2 (20.0) |
| *Pseudomonas aeruginosa* | 12 (5) | 7 (5.0) | 4 (6.6) | 1 (2.6) | 2 (5) | 1 (3.6) | 0 (0) | 1 (10.0) |
| *Escherichia coli* | 8 (3.4) | 4 (2.9) | 3 (4.9) | 1 (2.6) | 0 (0) | 0 (0) | 0 (0) | 0 (0) |
| *Serratia marcescens* | 4 (1.7) | 2 (1.4) | 0 (0) | 2 (5.3) | 0 (0) | 0 (0) | 0 (0) | 0 (0) |
| *Enterobacter cloacae* | 2 (0.8) | 1 (0.7) | 1 (1.6) | 0 (0) | 0 (0) | 0 (0) | 0 (0) | 0 (0) |
| *Escherichia coli/Shigella spp.* | 1 (0.4) | 1 (0.7) | 0 (0) | 0 (0) | 1 (2.5) | 0 (0) | 0 (0) | 1 (10.0) |
| *Klebsiella oxytoca* | 2 (0.8) | 1 (0.7) | 0 (0) | 1 (2.6) | 0 (0) | 0 (0) | 0 (0) | 0 (0) |
| *Morganella morganii* | 1 (0.4) | 1 (0.7) | 0 (0) | 0 (0) | 1 (2.5) | 0 (0) | 0 (0) | 1 (10.0) |
| *Neisseria meningitidis* | 2 (0.8) | 1 (0.7) | 1 (1.6) | 0 (0) | 0 (0) | 0 (0) | 0 (0) | 0 (0) |
| *Salmonella spp.* | 2 (0.8) | 1 (0.7) | 1 (1.6) | 0 (0) | 0 (0) | 0 (0) | 0 (0) | 0 (0) |
| **Gram positive bacteria** | 25 (10.5) | **17 (12.2)** | **2 (3.3)** | **6 (15.8)** | 9 (22.5) | **6 (21.4)** | **1 (50.0)** | **2 (20.0)** |
| *Staphylococcus aureus* | 5 (2.1) | 4 (2.9) | 0 (0) | 1 (2.6) | 3 (7.5) | 3 (10.7) | 0 (0) | 0 (0) |
| *Streptococcus pneumoniae* | 5 (2.1) | 4 (2.9) | 1 (1.6) | 0 (0) | 3 (7.5) | 1 (3.6) | 1 (50.0) | 1 (10.0) |
| *Streptococcus agalactiae* | 6 (2.5) | 3 (2.2) | 0 (0) | 3 (7.9) | 0 (0) | 0 (0) | 0 (0) | 0 (0) |
| *Enterococcus faecium* | 4 (1.7) | 2 (1.4) | 0 (0) | 2 (5.3) | 0 (0) | 0 (0) | 0 (0) | 0 (0) |
| *Enterococcus faecalis* | 1 (0.4) | 1 (0.7) | 0 (0) | 0 (0) | 1 (2.5) | 0 (0) | 0 (0) | 1 (10.0) |
| *Micrococcus species* | 2 (0.8) | 1 (0.7) | 1 (1.6) | 0 (0) | 0 (0) | 0 (0) | 0 (0) | 0 (0) |
| *Staphylococcus haemolyticus* | 1 (0.4) | 1 (0.7) | 0 (0) | 0 (0) | 1 (2.5) | 1 (3.6) | 0 (0) | 0 (0) |
| *Streptococcus pyogenes* | 1 (0.4) | 1 (0.7) | 0 (0) | 0 (0) | 1 (2.5) | 1 (3.6) | 0 (0) | 0 (0) |
| *Streptococcus spp.* | 2 (0.8) | 1 (0.7) | 1 (1.6) | 0 (0) | 0 (0) | 0 (0) | 0 (0) | 0 (0) |
| **Virus** | 1 (0.4) | **1 (0.7)** | **0 (0)** | **0 (0)** | 1 (2.5) | **0 (0)** | **1 (50.0)** | **0 (0)** |
| Adenovirus | 1 (0.4) | 1 (0.7) | 0 (0) | 0 (0) | 1 (2.5) | 0 (0) | 1 (50.0) | 0 (0) |
| **Fungus** | 12 (5) | **8 (5.8)** | **0 (0)** | **4 (10.5)** | 4 (10) | **4 (14.3)** | **0 (0)** | **0 (0)** |
| *Candida albicans* | 6 (2.5) | 4 (2.9) | 0 (0) | 2 (5.3) | 2 (5) | 2 (7.1) | 0 (0) | 0 (0) |
| *Candida glabrata* | 4 (1.7) | 2 (1.4) | 0 (0) | 2 (5.3) | 0 (0) | 0 (0) | 0 (0) | 0 (0) |
| *Candida parapsilosis* | 2 (0.8) | 2 (1.4) | 0 (0) | 0 (0) | 2 (5) | 2 (7.1) | 0 (0) | 0 (0) |
| **Parasite** | 1 (0.4) | **1 (0.7)** | **0 (0)** | **0 (0)** | 1 (2.5) | **0 (0)** | **0 (0)** | **1 (10.0)** |
| *Toxoplasma gondii* | 1 (0.4) | 1 (0.7) | 0 (0) | 0 (0) | 1 (2.5) | 0 (0) | 0 (0) | 1 (10.0) |
| **Polymicrobial** | 44 (18.5) | **26 (18.7)** | **12 (19.7)** | **6 (15.8)** | 8 (20) | **4 (14.3)** | **1 (50.0)** | **3 (30.0)** |
| **Number of cases with only one pathogen implicated** | 177 (74.4) | **102 (93.4)** | 45 (73.8) | 30 (78.9) | 27 (67.5) | 20 (71.4) | **1 (50.0)** | 6 (60.0) |
| **Number of cases with 2 pathogens implicated** | 32 (13.4) | **20 (14.4)** | 8 (13.1) | 4 (10.5) | 8 (20) | 4 (14.3) | **1 (50.0)** | 3 (30.0) |
| **Number of cases with 3 pathogens implicated** | 12 (5) | **6 (4.3)** | 4 (6.6) | 2 (5.3) | 0 (0) | **0 (0)** | **0 (0)** | **0 (0)** |
| **No pathogen detected** | 17 (7.1) | **11 (7.9)** | 4 (6.6) | 2 (5.3) | 5 (12.5) | 4 (14.3) | **0 (0)** | 1 (10.0) |

| **Table S9. Pathogen profile by age group for community-associated meningitis, CHAMPS, December 2016 – December 2023** | | | | | | | | |
| --- | --- | --- | --- | --- | --- | --- | --- | --- |
| **Pathogens** | **Total neonates** | Death in first 24 hours | Early neonate (1 to 6 days) | Late Neonate (7 to 27 days) | **Total infants and children** | Early infant (28 days-<6 months) | Late infant (6-12 months) | Child (12-59 months) |
|  | N = 79 | N = 20 | N = 28 | N = 31 | N = 52 | N = 18 | N = 14 | N = 20 |
| **Gram negative bacteria** | **55 (69.6)** | **9 (45.0)** | **24 (85.7)** | **22 (71.0)** | **21 (40.4)** | **11 (61.1)** | **3 (21.4)** | **7 (35.0)** |
| *Klebsiella pneumoniae* | 28 (35.4) | 8 (40.0) | 12 (42.9) | 8 (25.8) | 10 (19.2) | 5 (27.8) | 2 (14.3) | 3 (15.0) |
| *Escherichia coli* | 11 (13.9) | 3 (15.0) | 5 (17.9) | 3 (9.7) | 3 (5.8) | 2 (11.1) | 0 (0) | 1 (5.0) |
| *Acinetobacter baumannii* | 11 (13.9) | 0 (0) | 5 (17.9) | 6 (19.4) | 2 (3.8) | 2 (11.1) | 0 (0) | 0 (0) |
| *Pseudomonas aeruginosa* | 6 (7.6) | 1 (5.0) | 2 (7.1) | 3 (9.7) | 1 (1.9) | 0 (0) | 0 (0) | 1 (5.0) |
| *Salmonella spp.* | 1 (1.3) | 0 (0) | 0 (0) | 1 (3.2) | 3 (5.8) | 1 (5.6) | 0 (0) | 2 (10.0) |
| *NontypeableHaemophilus influenzae* | 0 (0) | 0 (0) | 0 (0) | 0 (0) | 2 (3.8) | 0 (0) | 0 (0) | 2 (10.0) |
| *Haemophilus influenzae Type A* | 1 (1.3) | 0 (0) | 1 (3.6) | 0 (0) | 1 (1.9) | 1 (5.6) | 0 (0) | 0 (0) |
| *Haemophilus influenzae Type B* | 0 (0) | 0 (0) | 0 (0) | 0 (0) | 2 (3.8) | 1 (5.6) | 1 (7.1) | 0 (0) |
| *Neisseria meningitidis* | 1 (1.3) | 0 (0) | 0 (0) | 1 (3.2) | 1 (1.9) | 0 (0) | 0 (0) | 1 (5.0) |
| *Citrobacter freundii* | 1 (1.3) | 0 (0) | 0 (0) | 1 (3.2) | 0 (0) | 0 (0) | 0 (0) | 0 (0) |
| *Escherichia coli/Shigella spp.* | 1 (1.3) | 0 (0) | 0 (0) | 1 (3.2) | 0 (0) | 0 (0) | 0 (0) | 0 (0) |
| *Klebsiella spp.* | 1 (1.3) | 0 (0) | 1 (3.6) | 0 (0) | 0 (0) | 0 (0) | 0 (0) | 0 (0) |
| *Orientia tsutsugamushi* | 1 (1.3) | 0 (0) | 0 (0) | 1 (3.2) | 0 (0) | 0 (0) | 0 (0) | 0 (0) |
| *Proteus mirabilis* | 1 (1.3) | 0 (0) | 0 (0) | 1 (3.2) | 0 (0) | 0 (0) | 0 (0) | 0 (0) |
| *Vibrio cholerae* | 1 (1.3) | 0 (0) | 0 (0) | 1 (3.2) | 0 (0) | 0 (0) | 0 (0) | 0 (0) |
| **Gram positive bacteria** | **18 (22.8)** | **9 (45.0)** | **1 (3.6)** | **8 (25.8)** | **25 (48.1)** | **8 (44.4)** | **8 (57.1)** | **9 (45.0)** |
| *Streptococcus pneumoniae* | 2 (2.5) | 2 (10.0) | 0 (0) | 0 (0) | 18 (34.6) | 6 (33.3) | 6 (42.9) | 6 (30.0) |
| *Streptococcus agalactiae* | 9 (11.4) | 7 (35.0) | 0 (0) | 2 (6.5) | 2 (3.8) | 0 (0) | 1 (7.1) | 1 (5.0) |
| *Staphylococcus aureus* | 3 (3.8) | 0 (0) | 0 (0) | 3 (9.7) | 1 (1.9) | 0 (0) | 0 (0) | 1 (5.0) |
| *Streptococcus spp.* | 0 (0) | 0 (0) | 0 (0) | 0 (0) | 3 (5.8) | 1 (5.6) | 1 (7.1) | 1 (5.0) |
| *Enterococcus faecalis* | 2 (2.5) | 0 (0) | 0 (0) | 2 (6.5) | 0 (0) | 0 (0) | 0 (0) | 0 (0) |
| *Enterococcus faecium* | 1 (1.3) | 0 (0) | 0 (0) | 1 (3.2) | 0 (0) | 0 (0) | 0 (0) | 0 (0) |
| *Stenotrophomonas maltophilia* | 1 (1.3) | 0 (0) | 1 (3.6) | 0 (0) | 0 (0) | 0 (0) | 0 (0) | 0 (0) |
| *Streptococcus pyogenes* | 0 (0) | 0 (0) | 0 (0) | 0 (0) | 1 (1.9) | 1 (5.6) | 0 (0) | 0 (0) |
| **Virus** | **1 (1.3)** | **0 (0)** | **0 (0)** | **1 (3.2)** | **1 (1.9)** | **1 (5.6)** | **0 (0)** | **0 (0)** |
| Cytomegalovirus | 0 (0) | 0 (0) | 0 (0) | 0 (0) | 1 (1.9) | 1 (5.6) | 0 (0) | 0 (0) |
| Parechovirus | 1 (1.3) | 0 (0) | 0 (0) | 1 (3.2) | 0 (0) | 0 (0) | 0 (0) | 0 (0) |
| **Fungus** | **1 (1.3)** | **0 (0)** | **0 (0)** | **1 (3.2)** | **0 (0)** | **0 (0)** | **0 (0)** | **0 (0)** |
| *Candida albicans* | 1 (1.3) | 0 (0) | 0 (0) | 1 (3.2) | 0 (0) | 0 (0) | 0 (0) | 0 (0) |
| **Parasite** | **0 (0)** | **0 (0)** | **0 (0)** | **0 (0)** | **0 (0)** | **0 (0)** | **0 (0)** | **0 (0)** |
| **Polymicrobial** | **11 (13.9)** | **3 (15.0)** | **2 (7.1)** | **6 (19.4)** | **8 (15.4)** | **4 (22.2)** | **1 (7.1)** | **3 (15.0)** |
| **Number of cases with only one pathogen implicated** | **60 (75.9)** | **15 (75.0)** | **23 (82.1)** | **22 (71.0)** | **34 (65.4)** | **13 (72.2)** | **9 (64.3)** | **12 (60.0)** |
| **Number of cases with 2 pathogens implicated** | **8 (10.1)** | **3 (15.0)** | **2 (7.1)** | **3 (9.7)** | **7 (13.5)** | **4 (22.2)** | **1 (7.1)** | **2 (10.0)** |
| **Number of cases with 3 pathogens implicated** | **3 (3.8)** | **0 (0)** | **0 (0)** | **3 (9.7)** | **1 (1.9)** | **0 (0)** | **0 (0)** | **1 (5.0)** |
| **No pathogen detected** | **9 (11.4)** | **2 (10.0)** | **3 (9.7)** | **4 (12.5)** | **10 (19.2)** | **1 (5.6)** | **4 (28.6)** | **5 (25.0)** |

| **Table S10. Pathogens implicated in congenital/perinatal infection and/or sepsis for deaths with community- or hospital-associated meningitis by age group, CHAMPS, December 2016 – December 2023** | | | | |
| --- | --- | --- | --- | --- |
|  | **Neonates** | | **Infants and children** | |
| Pathogen | Community (N = 63) | Facility (N = 87) | Community (N = 37) | Facility (N = 31) |
| *Acinetobacter baumannii* | 11 (17.5) | 54 (62.1) | 1 (2.7) | 13 (41.9) |
| *Bordetella spp.* | 1 (1.6) | 0 (0) | 0 (0) | 0 (0) |
| *Candida albicans* | 0 (0) | 2 (2.3) | 1 (2.7) | 2 (6.5) |
| *Candida auris* | 2 (3.2) | 0 (0) | 0 (0) | 0 (0) |
| *Candida glabrata* | 0 (0) | 2 (2.3) | 0 (0) | 0 (0) |
| *Candida parapsilosis* | 0 (0) | 1 (1.1) | 1 (2.7) | 1 (3.2) |
| Candida spp. | 0 (0) | 0 (0) | 0 (0) | 1 (3.2) |
| *Citrobacter freundii* | 1 (1.6) | 0 (0) | 0 (0) | 0 (0) |
| Cytomegalovirus | 1 (1.6) | 0 (0) | 3 (8.1) | 0 (0) |
| *Enterobacter cloacae* | 0 (0) | 2 (2.3) | 0 (0) | 0 (0) |
| *Enterococcus faecalis* | 0 (0) | 2 (2.3) | 0 (0) | 2 (6.5) |
| *Enterococcus faecium* | 3 (4.8) | 7 (8.0) | 1 (2.7) | 2 (6.5) |
| Enterovirus | 0 (0) | 1 (1.1) | 0 (0) | 0 (0) |
| *Escherichia coli* | 8 (12.7) | 4 (4.6) | 9 (24.3) | 3 (9.7) |
| *Escherichia coli/Shigella spp.* | 0 (0) | 0 (0) | 1 (2.7) | 0 (0) |
| *Haemophilus influenzae* Type A | 1 (1.6) | 0 (0) | 2 (5.4) | 0 (0) |
| *Haemophilus influenzae* Type B | 0 (0) | 0 (0) | 2 (5.4) | 0 (0) |
| *Klebsiella oxytoca* | 0 (0) | 2 (2.3) | 0 (0) | 0 (0) |
| *Klebsiella pneumoniae* | 32 (50.8) | 48 (55.2) | 12 (32.4) | 23 (74.2) |
| *Klebsiella terragina* | 0 (0) | 1 (1.1) | 0 (0) | 0 (0) |
| *Listeria monocytogenes* | 0 (0) | 1 (1.1) | 0 (0) | 0 (0) |
| *Morganella morganii* | 0 (0) | 1 (1.1) | 0 (0) | 1 (3.2) |
| *Neisseria meningitidis* | 1 (1.6) | 1 (1.1) | 0 (0) | 0 (0) |
| *Orientia tsutsugamushi* | 1 (1.6) | 0 (0) | 0 (0) | 0 (0) |
| *Pneumocystis jirovecii* | 0 (0) | 0 (0) | 0 (0) | 1 (3.2) |
| *Proteus mirabilis* | 1 (1.6) | 0 (0) | 0 (0) | 0 (0) |
| *Proteus vulgaris* | 0 (0) | 1 (1.1) | 0 (0) | 0 (0) |
| *Pseudomonas aeruginosa* | 6 (9.5) | 6 (6.9) | 1 (2.7) | 1 (3.2) |
| *Salmonella spp.* | 2 (3.2) | 1 (1.1) | 3 (8.1) | 0 (0) |
| *Serratia marcescens* | 0 (0) | 1 (1.1) | 0 (0) | 0 (0) |
| Serratia spp. | 0 (0) | 0 (0) | 1 (2.7) | 0 (0) |
| *Staphylococcus aureus* | 2 (3.2) | 3 (3.4) | 1 (2.7) | 5 (16.1) |
| *Streptococcus agalactiae* | 7 (11.1) | 6 (6.9) | 1 (2.7) | 0 (0) |
| *Streptococcus pneumoniae* | 2 (3.2) | 0 (0) | 14 (37.8) | 4 (12.9) |
| Streptococcus pyogenes | 0 (0) | 0 (0) | 1 (2.7) | 0 (0) |
| *Streptococcus spp.* | 0 (0) | 1 (1.1) | 4 (10.8) | 0 (0) |
| *Vibrio cholerae* | 1 (1.6) | 0 (0) | 0 (0) | 0 (0) |

| **Table S11. Pathogen profile for community- and hospital-associated meningitis by sites, CHAMPS, December 2016 – December 2023** | | | | | | | | | | | | | | |
| --- | --- | --- | --- | --- | --- | --- | --- | --- | --- | --- | --- | --- | --- | --- |
|  | **Community-associated meningitis** | | | | | | | **Hospital-associated meningitis** | | | | | | |
| **Pathogens** | Bangladesh | Ethiopia | Kenya | Mali | Mozambique | Sierra leone | South Africa | Bangladesh | Ethiopia | Kenya | Mali | Mozambique | Sierra leone | South Africa |
|  | N = 7 | N = 37 | N = 5 | N = 12 | N = 11 | N = 17 | N = 42 | N = 2 | N = 15 | N = 0 | N = 6 | N = 2 | N = 6 | N = 108 |
| **Gram negative bacteria** | **5 (71.4)** | **24 (64.9)** | **3 (60.0)** | **8 (66.7)** | **3 (27.3)** | **11 (64.7)** | **22 (52.4)** | **2 (100.0)** | **14 (93.3)** | **0 (0)** | **3 (50.0)** | **2 (100.0)** | **5 (83.3)** | **84 (77.8)** |
| *Klebsiella pneumoniae* | 1 (14.3) | 19 (51.4) | 2 (40.0) | 2 (16.7) | 0 (0) | 6 (35.3) | 8 (19.0) | 2 (100.0) | 13 (86.7) | 2 (33.3) | 0 (0) | 1 (50.0) | 5 (83.3) | 34 (31.5) |
| *Acinetobacter baumannii* | 1 (14.3) | 1 (2.7) | 0 (0) | 0 (0) | 0 (0) | 1 (5.9) | 10 (23.8) | 0 (0) | 0 (0) | 0 (0) | 0 (0) | 1 (50.0) | 0 (0) | 55 (50.9) |
| *Escherichia coli* | 0 (0) | 5 (13.5) | 0 (0) | 2 (16.7) | 1 (9.1) | 2 (11.8) | 4 (9.5) | 0 (0) | 2 (13.3) | 0 (0) | 0 (0) | 0 (0) | 0 (0) | 2 (1.9) |
| *Pseudomonas aeruginosa* | 0 (0) | 4 (10.8) | 0 (0) | 1 (8.3) | 1 (9.1) | 0 (0) | 1 (2.4) | 0 (0) | 4 (26.7) | 0 (0) | 0 (0) | 0 (0) | 0 (0) | 3 (2.8) |
| *Salmonella spp.* | 0 (0) | 1 (2.7) | 0 (0) | 2 (16.7) | 0 (0) | 1 (5.9) | 0 (0) | 0 (0) | 1 (6.7) | 0 (0) | 0 (0) | 0 (0) | 0 (0) | 0 (0) |
| *Neisseria meningitidis* | 1 (14.3) | 0 (0) | 0 (0) | 0 (0) | 0 (0) | 0 (0) | 1 (2.4) | 0 (0) | 1 (6.7) | 0 (0) | 0 (0) | 0 (0) | 0 (0) | 0 (0) |
| *Escherichia coli/Shigella spp.* | 0 (0) | 1 (2.7) | 0 (0) | 0 (0) | 0 (0) | 0 (0) | 0 (0) | 0 (0) | 0 (0) | 1 (16.7) | 0 (0) | 0 (0) | 0 (0) | 0 (0) |
| *Haemophilus influenzae* | 0 (0) | 1 (2.7) | 0 (0) | 0 (0) | 0 (0) | 0 (0) | 1 (2.4) | 0 (0) | 0 (0) | 0 (0) | 0 (0) | 0 (0) | 0 (0) | 0 (0) |
| *Haemophilus influenzae Type A* | 0 (0) | 1 (2.7) | 0 (0) | 0 (0) | 1 (9.1) | 0 (0) | 0 (0) | 0 (0) | 0 (0) | 0 (0) | 0 (0) | 0 (0) | 0 (0) | 0 (0) |
| *Haemophilus influenzae Type B* | 0 (0) | 0 (0) | 0 (0) | 2 (16.7) | 0 (0) | 0 (0) | 0 (0) | 0 (0) | 0 (0) | 0 (0) | 0 (0) | 0 (0) | 0 (0) | 0 (0) |
| *Serratia marcescens* | 0 (0) | 0 (0) | 0 (0) | 0 (0) | 0 (0) | 0 (0) | 0 (0) | 0 (0) | 0 (0) | 0 (0) | 0 (0) | 0 (0) | 0 (0) | 2 (1.9) |
| *Citrobacter freundii* | 0 (0) | 0 (0) | 1 (20.0) | 0 (0) | 0 (0) | 0 (0) | 0 (0) | 0 (0) | 0 (0) | 0 (0) | 0 (0) | 0 (0) | 0 (0) | 0 (0) |
| *Enterobacter cloacae* | 0 (0) | 0 (0) | 0 (0) | 0 (0) | 0 (0) | 0 (0) | 0 (0) | 0 (0) | 1 (6.7) | 0 (0) | 0 (0) | 0 (0) | 0 (0) | 0 (0) |
| *Klebsiella oxytoca* | 0 (0) | 0 (0) | 0 (0) | 0 (0) | 0 (0) | 0 (0) | 0 (0) | 0 (0) | 0 (0) | 0 (0) | 0 (0) | 0 (0) | 0 (0) | 1 (0.9) |
| *Klebsiella spp.* | 0 (0) | 0 (0) | 0 (0) | 0 (0) | 0 (0) | 1 (5.9) | 0 (0) | 0 (0) | 0 (0) | 0 (0) | 0 (0) | 0 (0) | 0 (0) | 0 (0) |
| *Morganella morganii* | 0 (0) | 0 (0) | 0 (0) | 0 (0) | 0 (0) | 0 (0) | 0 (0) | 0 (0) | 0 (0) | 0 (0) | 0 (0) | 0 (0) | 0 (0) | 1 (0.9) |
| *Orientia tsutsugamushi* | 1 (14.3) | 0 (0) | 0 (0) | 0 (0) | 0 (0) | 0 (0) | 0 (0) | 0 (0) | 0 (0) | 0 (0) | 0 (0) | 0 (0) | 0 (0) | 0 (0) |
| *Proteus mirabilis* | 1 (14.3) | 0 (0) | 0 (0) | 0 (0) | 0 (0) | 0 (0) | 0 (0) | 0 (0) | 0 (0) | 0 (0) | 0 (0) | 0 (0) | 0 (0) | 0 (0) |
| *Vibrio cholerae* | 0 (0) | 1 (2.7) | 0 (0) | 0 (0) | 0 (0) | 0 (0) | 0 (0) | 0 (0) | 0 (0) | 0 (0) | 0 (0) | 0 (0) | 0 (0) | 0 (0) |
| **Gram positive bacteria** | **2 (28.6)** | **8 (21.6)** | **3 (60.0)** | **5 (41.7)** | **6 (54.5)** | **2 (11.8)** | **17 (40.5)** | **0 (0)** | **1 (6.7)** | **0 (0)** | **1 (16.7)** | **0 (0)** | **1 (16.7)** | **14 (13.0)** |
| *Streptococcus pneumoniae* | 0 (0) | 3 (8.1) | 2 (40.0) | 2 (16.7) | 6 (54.5) | 1 (5.9) | 6 (14.3) | 0 (0) | 0 (0) | 1 (16.7) | 0 (0) | 0 (0) | 0 (0) | 3 (2.8) |
| *Streptococcus agalactiae* | 0 (0) | 1 (2.7) | 1 (20.0) | 1 (8.3) | 0 (0) | 1 (5.9) | 7 (16.7) | 0 (0) | 0 (0) | 0 (0) | 0 (0) | 0 (0) | 0 (0) | 3 (2.8) |
| *Staphylococcus aureus* | 1 (14.3) | 2 (5.4) | 0 (0) | 0 (0) | 0 (0) | 0 (0) | 1 (2.4) | 0 (0) | 0 (0) | 0 (0) | 0 (0) | 0 (0) | 0 (0) | 4 (3.7) |
| *Streptococcus spp.* | 0 (0) | 0 (0) | 0 (0) | 2 (16.7) | 0 (0) | 0 (0) | 1 (2.4) | 0 (0) | 1 (6.7) | 0 (0) | 0 (0) | 0 (0) | 0 (0) | 0 (0) |
| *Enterococcus faecalis* | 0 (0) | 0 (0) | 0 (0) | 0 (0) | 0 (0) | 0 (0) | 2 (4.8) | 0 (0) | 0 (0) | 0 (0) | 0 (0) | 0 (0) | 0 (0) | 1 (0.9) |
| *Enterococcus faecium* | 1 (14.3) | 0 (0) | 0 (0) | 0 (0) | 0 (0) | 0 (0) | 0 (0) | 0 (0) | 0 (0) | 0 (0) | 0 (0) | 0 (0) | 0 (0) | 2 (1.9) |
| *Streptococcus pyogenes* | 0 (0) | 1 (2.7) | 0 (0) | 0 (0) | 0 (0) | 0 (0) | 0 (0) | 0 (0) | 0 (0) | 0 (0) | 0 (0) | 0 (0) | 0 (0) | 1 (0.9) |
| *Micrococcus species* | 0 (0) | 0 (0) | 0 (0) | 0 (0) | 0 (0) | 0 (0) | 0 (0) | 0 (0) | 1 (6.7) | 0 (0) | 0 (0) | 0 (0) | 0 (0) | 0 (0) |
| *Staphylococcus haemolyticus* | 0 (0) | 0 (0) | 0 (0) | 0 (0) | 0 (0) | 0 (0) | 0 (0) | 0 (0) | 0 (0) | 0 (0) | 0 (0) | 0 (0) | 1 (16.7) | 0 (0) |
| *Stenotrophomonas maltophilia* | 0 (0) | 1 (2.7) | 0 (0) | 0 (0) | 0 (0) | 0 (0) | 0 (0) | 0 (0) | 0 (0) | 0 (0) | 0 (0) | 0 (0) | 0 (0) | 0 (0) |
| **Viruses** | **0 (0)** | **0 (0)** | **0 (0)** | **0 (0)** | **0 (0)** | **1 (5.9)** | **1 (2.4)** | **0 (0)** | **0 (0)** | **0 (0)** | **0 (0)** | **0 (0)** | **0 (0)** | **1 (0.9)** |
| Adenovirus | 0 (0) | 0 (0) | 0 (0) | 0 (0) | 0 (0) | 0 (0) | 0 (0) | 0 (0) | 0 (0) | 0 (0) | 0 (0) | 0 (0) | 0 (0) | 1 (0.9) |
| Cytomegalovirus | 0 (0) | 0 (0) | 0 (0) | 0 (0) | 0 (0) | 1 (5.9) | 0 (0) | 0 (0) | 0 (0) | 0 (0) | 0 (0) | 0 (0) | 0 (0) | 0 (0) |
| Parechovirus | 0 (0) | 0 (0) | 0 (0) | 0 (0) | 0 (0) | 0 (0) | 1 (2.4) | 0 (0) | 0 (0) | 0 (0) | 0 (0) | 0 (0) | 0 (0) | 0 (0) |
| **Fungi** | **0 (0)** | **0 (0)** | **0 (0)** | **0 (0)** | **0 (0)** | **0 (0)** | **1 (2.4)** | **0 (0)** | **0 (0)** | **0 (0)** | **0 (0)** | **0 (0)** | **0 (0)** | **8 (7.4)** |
| *Candida albicans* | 0 (0) | 0 (0) | 0 (0) | 0 (0) | 0 (0) | 0 (0) | 1 (2.4) | 0 (0) | 0 (0) | 0 (0) | 0 (0) | 0 (0) | 0 (0) | 4 (3.7) |
| *Candida glabrata* | 0 (0) | 0 (0) | 0 (0) | 0 (0) | 0 (0) | 0 (0) | 0 (0) | 0 (0) | 0 (0) | 0 (0) | 0 (0) | 0 (0) | 0 (0) | 2 (1.9) |
| *Candida parapsilosis* | 0 (0) | 0 (0) | 0 (0) | 0 (0) | 0 (0) | 0 (0) | 0 (0) | 0 (0) | 0 (0) | 0 (0) | 0 (0) | 0 (0) | 0 (0) | 2 (1.9) |
| **Parasites** | **0 (0)** | **0 (0)** | **0 (0)** | **0 (0)** | **0 (0)** | **0 (0)** | **0 (0)** | **0 (0)** | **0 (0)** | **0 (0)** | **0 (0)** | **0 (0)** | **1 (16.7)** | **0 (0)** |
| *Toxoplasma gondii* | 0 (0) | 0 (0) | 0 (0) | 0 (0) | 0 (0) | 0 (0) | 0 (0) | 0 (0) | 0 (0) | 0 (0) | 0 (0) | 0 (0) | 1 (16.7) | 0 (0) |
| **Polymicrobial** | **0 (0)** | **9 (24.3)** | **1 (20.0)** | **2 (16.7)** | **1 (9.1)** | **1 (5.9)** | **5 (11.9)** | **0 (0)** | **6 (40.0)** | **0 (0)** | **0 (0)** | **0 (0)** | **1 (16.7)** | **19 (17.6)** |
| **No pathogen detected** | **0 (0)** | **7 (18.9)** | **0 (0)** | **0 (0)** | **3 (27.3)** | **4 (23.5)** | **4 (9.5)** | **0 (0)** | **1 (6.7)** | **0 (0)** | **2 (33.3)** | **0 (0)** | **0 (0)** | **8 (7.4)** |
| **Number of cases with only one pathogen implicated** | **7 (100.0)** | **21 (56.8)** | **4 (80.0)** | **10 (83.3)** | **7 (63.6)** | **12 (70.6)** | **33 (78.6)** | **2 (100.0)** | **8 (53.3)** | **0 (0)** | **4 (66.7)** | **2 (100.0)** | **5 (83.3)** | **81 (75.0)** |
| **Number of cases with 2 pathogens implicated** | **0 (0)** | **6 (16.2)** | **1 (20.0)** | **2 (16.7)** | **1 (9.1)** | **1 (5.9)** | **4 (9.5)** | **0 (0)** | **2 (13.3)** | **0 (0)** | **0 (0)** | **0 (0)** | **1 (16.7)** | **17 (15.7)** |
| **Number of cases with 3 pathogens implicated** | **0 (0)** | **3 (8.1)** | **0 (0)** | **0 (0)** | **0 (0)** | **0 (0)** | **1 (2.4)** | **0 (0)** | **4 (26.7)** | **0 (0)** | **0 (0)** | **0 (0)** | **0 (0)** | **2 (1.9)** |
| **Median number of pathogens implicated per case (IQR)** | **1 (1, 1)** | **1 (1, 2)** | **1 (1, 1)** | **1 (1, 1)** | **1 (1, 1)** | **1 (1, 1)** | **1 (1, 1)** | **1 (1, 1)** | **1 (1, 3)** | **--** | **1 (1, 1)** | **1 (1, 1)** | **1 (1, 1)** | **1 (1, 1)** |

| **Table S12**. Minimally invasive tissue sampling (MITS) procedures that were deemed essential for determining causes of death by age group for deaths with meningitis in the causal chain, CHAMPS, December 2016 – December 2023 |  |  |  |  |  |  |  |  |
| --- | --- | --- | --- | --- | --- | --- | --- | --- |
|  | **Total neonates** | Death in first 24 hours | Early neonate (1 to 6 days) | Late Neonate (7 to 27 days) | **Total infants and children** | Early infant (28 days-<6 months) | Late infant (6-<12 months) | Child (12-59 months) |
| Procedure | **N = 178** | N = 20 | N = 89 | N = 69 | **N = 92** | N = 46 | N = 16 | N = 30 |
| Case clinical data - including antemortem diagnostic data | **156 (87.6)** | 12 (60.0) | 80 (89.9) | 64 (92.8) | **72 (78.3)** | 35 (76.1) | 13 (81.2) | 24 (80.0) |
| Case postmortem microbiology/culture | **159 (89.3)** | 13 (65.0) | 84 (94.4) | 62 (89.9) | **65 (70.7)** | 35 (76.1) | 12 (75.0) | 18 (60.0) |
| Case postmortem molecular diagnostics (TAC) | **150 (84.3)** | 16 (80.0) | 77 (86.5) | 57 (82.6) | **73 (79.3)** | 37 (80.4) | 12 (75.0) | 24 (80.0) |
| Pathology- local final report | **138 (77.5)** | 17 (85.0) | 71 (79.8) | 50 (72.5) | **74 (80.4)** | 40 (87.0) | 13 (81.2) | 21 (70.0) |
| Pathology- CPL diagnosis | **118 (66.3)** | 15 (75.0) | 58 (65.2) | 45 (65.2) | **67 (72.8)** | 34 (73.9) | 14 (87.5) | 19 (63.3) |
| Pathology- CPL immunohistochemistry | **71 (39.9)** | 5 (25.0) | 41 (46.1) | 25 (36.2) | **49 (53.3)** | 24 (52.2) | 11 (68.8) | 14 (46.7) |
| Maternal clinical data | **83 (46.6)** | 10 (50.0) | 49 (55.1) | 24 (34.8) | **9 (9.8)** | 7 (15.2) | 0 (0) | 2 (6.7) |
| MITS photography | **55 (30.9)** | 9 (45.0) | 31 (34.8) | 15 (21.7) | **29 (31.5)** | 13 (28.3) | 4 (25.0) | 12 (40.0) |
| Verbal autopsy narrative | **60 (33.7)** | 9 (45.0) | 32 (36.0) | 19 (27.5) | **31 (33.7)** | 14 (30.4) | 6 (37.5) | 11 (36.7) |
| Verbal autopsy list of symptoms/conditions | **54 (30.3)** | 9 (45.0) | 28 (31.5) | 17 (24.6) | **26 (28.3)** | 11 (23.9) | 5 (31.2) | 10 (33.3) |
| MITS measurements | **53 (29.8)** | 11 (55.0) | 29 (32.6) | 13 (18.8) | **25 (27.2)** | 10 (21.7) | 4 (25.0) | 11 (36.7) |
| Case postmortem HIV testing | **33 (18.5)** | 5 (25.0) | 21 (23.6) | 7 (10.1) | **21 (22.8)** | 8 (17.4) | 4 (25.0) | 9 (30.0) |
| Case postmortem TB testing | **36 (20.2)** | 6 (30.0) | 23 (25.8) | 7 (10.1) | **16 (17.4)** | 6 (13.0) | 4 (25.0) | 6 (20.0) |
| Case postmortem malaria testing | **38 (21.3)** | 8 (40.0) | 23 (25.8) | 7 (10.1) | **14 (15.2)** | 7 (15.2) | 3 (18.8) | 4 (13.3) |
| Pathology- CPL PCR | **17 (9.6)** | 2 (10.0) | 11 (12.4) | 4 (5.8) | **18 (19.6)** | 8 (17.4) | 5 (31.2) | 5 (16.7) |

| **Table S13. Number of meningitis deaths deemed preventable by age group, CHAMPS, December 2016 – December 2023** | | | | | | | |
| --- | --- | --- | --- | --- | --- | --- | --- |
|  | All | Death in first 24 hours | Early neonate (1 to 6 days) | Late Neonate (7 to 27 days) | Early infant (28 days-<6 months) | Late infant (6-<12 months) | Child (12-59 months) |
|  | N = 270 | N = 20 | N = 89 | N = 69 | N = 46 | N = 16 | N = 30 |
| Preventable | 219 (81.1) | 13 (65.0) | 77 (86.5) | 59 (85.5) | 34 (73.9) | 10 (62.5) | 26 (86.7) |
| Preventable under certain conditions | 10 (3.7) | 1 (5.0) | 3 (3.4) | 2 (2.9) | 2 (4.3) | 1 (6.2) | 1 (3.3) |
| Not preventable | 40 (14.8) | 6 (30.0) | 8 (9.0) | 8 (11.6) | 10 (21.7) | 5 (31.2) | 3 (10.0) |
| Not recorded | 1 (0.4) | 0 (0) | 1 (1.1) | 0 (0) | 0 (0) | 0 (0) | 0 (0) |

| **Table S14. Expert (DeCoDe) panel recommendations for preventing meningitis deaths, CHAMPS, December 2016 – December 2023** | | | | |  |  |  |
| --- | --- | --- | --- | --- | --- | --- | --- |
|  | All | Death in first 24 hours | Early neonate (1 to 6 days) | Late Neonate (7 to 27 days) | Early infant (28 days-<6 months) | Late infant (6-<12 months) | Child (12-59 months) |
|  | N = 220* | N = 13 | N = 81 | N = 57 | N = 33 | N = 10 | N = 26 |
| Improved ANC and obstetric care and management | 60 (27.3) | 9 (69.2) | 32 (39.5) | 13 (22.8) | 5 (15.2) | 0 (0) | 1 (3.8) |
| Improved clinical management and quality of care | 98 (44.5) | 11 (84.6) | 32 (39.5) | 14 (24.6) | 15 (45.5) | 6 (60.0) | 20 (76.9) |
| Improved family planning | 19 (8.6) | 4 (30.8) | 8 (9.9) | 4 (7.0) | 0 (0) | 2 (20.0) | 1 (3.8) |
| Improved health-seeking behavior | 59 (26.8) | 6 (46.2) | 15 (18.5) | 11 (19.3) | 13 (39.4) | 5 (50.0) | 9 (34.6) |
| Improved health education [immunizations, preventing malnutrition, diarrhea, burns, poisoning, etc.] | 28 (12.7) | 1 (7.7) | 2 (2.5) | 3 (5.3) | 8 (24.2) | 6 (60.0) | 8 (30.8) |
| Improved HIV prevention and control | 9 (4.1) | 0 (0) | 2 (2.5) | 0 (0) | 3 (9.1) | 2 (20.0) | 2 (7.7) |
| Improved infection prevention and control | 148 (67.3) | 6 (46.2) | 65 (80.2) | 46 (80.7) | 19 (57.6) | 3 (30.0) | 9 (34.6) |
| Improved nutritional support | 20 (9.1) | 3 (23.1) | 0 (0) | 3 (5.3) | 4 (12.1) | 3 (30.0) | 7 (26.9) |
| Improved transport system | 10 (4.5) | 2 (15.4) | 0 (0) | 1 (1.8) | 0 (0) | 3 (30.0) | 4 (15.4) |
| Improved vaccinations | 10 (4.5) | 0 (0) | 0 (0) | 0 (0) | 2 (6.1) | 4 (40.0) | 4 (15.4) |
| *Among deaths deemed preventable that had recommendations |  |  |  |  |  |  |  |

Supplemental Figure 1: Inclusion and exclusion criteria for minimally invasive tissue sampling (MITS) and non-MITS enrollment (ref Clin Infect Dis, Volume 69, Issue Supplement_4, 15 October 2019, Pages S262–S273, <https://doi.org/10.1093/cid/ciz599>


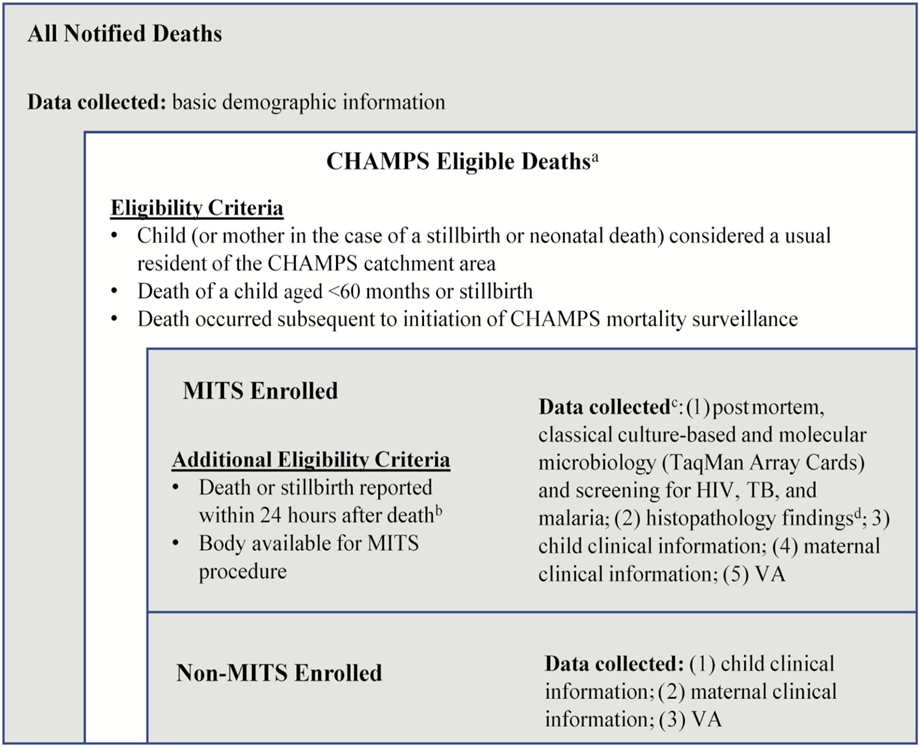


Footnote: ^a^small proportion of confirmed Child Health and Mortality Prevention Surveillance (CHAMPS) eligible deaths (ie, family was approached for eligibility screening and confirmed eligibility information) are not enrolled in CHAMPS due to parental nonconsent or loss to follow-up. ^b^The MITS timeframe may be extended up to 72 hours after death if body is refrigerated shortly after death. ^c^Circumstances may prevent the MITS from being conducted after MITS consent has been obtained. In these infrequent cases, data collection aligns with non-MITS procedures. ^d^Histology is conducted at the site and at the central pathology laboratory located at the US Centers for Disease Control and Prevention. Abbreviations: CHAMPS, Child Health and Mortality Prevention Surveillance; HIV, human immunodeficiency virus; MITS, minimally invasive tissue sampling; TB, tuberculosis; VA, verbal autopsy.

**Figure S2.** Frequencies of co-infections attributed to meningitis for neonates (A) and infants/children (B), CHAMPS, December 2016 – December 2023


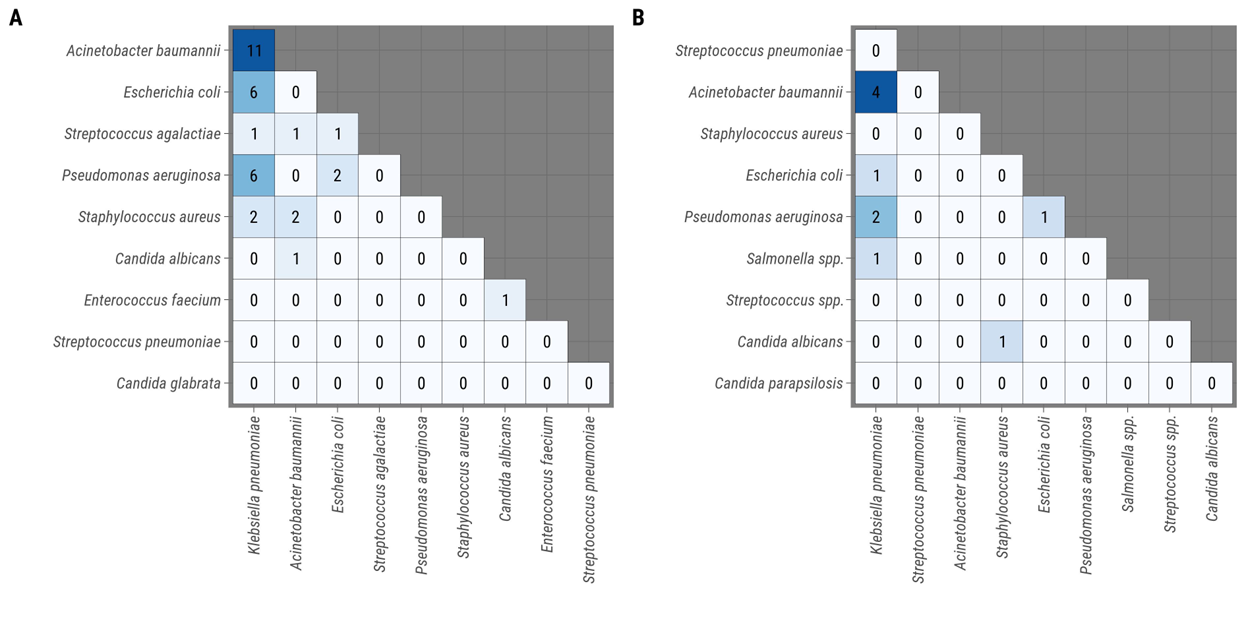


**Figure S3**. Meningitis mortality fractions from CHAMPS expert panel (DeCoDe), as well as interVA and inSilico verbal autopsy tools


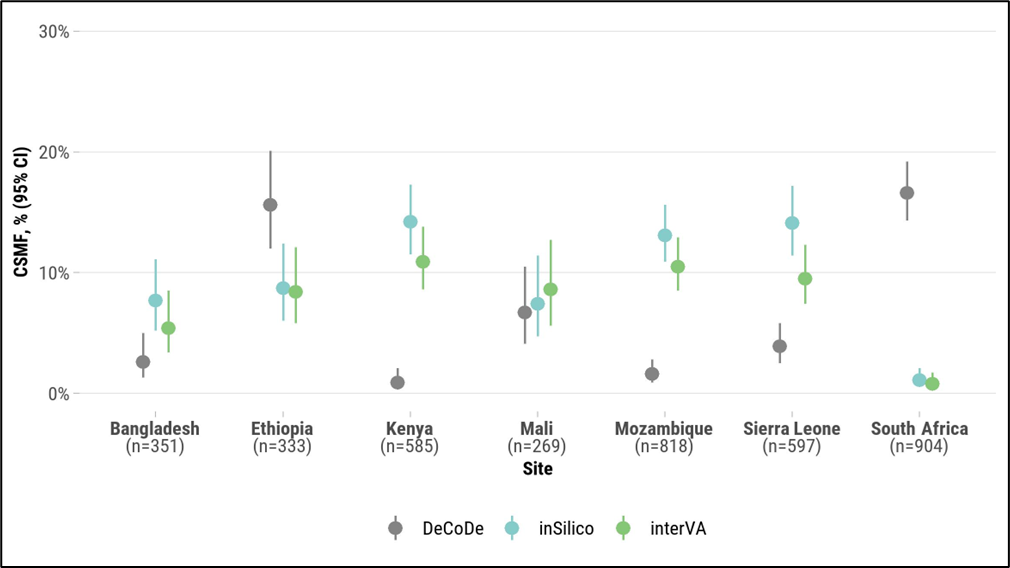


| **CHAMPS Consortium - DeCoDe Panelists or Other (Optional review)** | | | | | |
| --- | --- | --- | --- | --- | --- |
| Name | Academic Degrees | Institution | Email | Role or Contribution | Site |
| Sanwarul Bari | MD | Maternal and Child Health Division, International Center for Diarrhoeal Diseases Research (icddr,b), Dhaka, Bangladesh | [bari@icddrb.org](mailto:bari@icddrb.org) | Data collection | Bangladesh |
| Shahana Parveen | MSS | International Centre for Diarrhoeal Disease Research, Bangladesh (icddr,b) | [shahana@icddrb.org](mailto:shahana@icddrb.org) | Data collection | Bangladesh |
| Mohammed Kamal | PhD | Bangabandhu Sheikh Mujib Medical University, Dhaka, Bangladesh | [kamalzsr@yahoo.com; kamal.bsmmu@gmail.com](mailto:kamalzsr@yahoo.com;%20kamal.bsmmu@gmail.com) | Data collection | Bangladesh |
| A.S.M. Nawshad Uddin Ahmed | FCPS | Bangladesh Institute of Child Health at Dhaka University and Dhaka Shishu Children’s Hospital | [dr_nawshad@yahoo.com](mailto:dr_nawshad@yahoo.com) | Data collection | Bangladesh |
| Mahbubul Hoque | FCPS | Bangladesh Institute of Child Health at Dhaka University and Dhaka Shishu Children’s Hospital | [mahbubulhoque2013@gmail.com](mailto:mahbubulhoque2013@gmail.com) | Data collection | Bangladesh |
| Saria Tasnim | FCPS | Dhaka Community Medical College and Hospital | [sariatasnim2007@gmail.com](mailto:sariatasnim2007@gmail.com) | Data collection | Bangladesh |
| Ferdousi Islam | FCPS | Popular Medical College and Hospital in Dhaka, Bangladesh | [ferdousi.island23@gmail.com](mailto:ferdousi.island23@gmail.com) | Data collection | Bangladesh |
| Farida Ariuman | FCPS | National Institute of Cancer Research and Hospital (NICRH), Dhaka, Bangladesh | [drarju35cmc@gmail.com](mailto:drarju35cmc@gmail.com) | Data collection | Bangladesh |
| Mohammad Mosiur Rahman | MD | Bangabandhu Sheikh Mujib Medical University, Dhaka, Bangladesh | [mohammadmosiur76@gmail.com](mailto:mohammadmosiur76@gmail.com) | Data collection | Bangladesh |
| Ferdousi Begum | MD | Bangabandhu Sheikh Mujib Medical University (BSMMU) | [ferdousy_begum2000@yahoo.com](mailto:ferdousy_begum2000@yahoo.com) | Data collection | Bangladesh |
| K. Zaman | PhD | International Centre for Diarrhoeal Disease Research, Bangladesh (icddr,b) | [kzaman@icddrb.org](mailto:kzaman@icddrb.org) | Data collection | Bangladesh |
| Mustafizur Rahman | PhD | International Centre for Diarrhoeal Disease Research, Bangladesh (icddr,b) | [mustafizur@icddrb.org](mailto:mustafizur@icddrb.org) | Data collection | Bangladesh |
| Dilruba Ahmed | PhD | International Centre for Diarrhoeal Disease Research, Bangladesh (icddr,b) | [dahmed@icddrb.org](mailto:dahmed@icddrb.org) | Data collection | Bangladesh |
| Meerjady Sabrina Flora | PhD | Institute of Epidemiology, Disease Control, and Research (IEDCR), Dhaka, Bangladesh | [meerflora@yahoo.com](mailto:meerflora@yahoo.com) | Data collection | Bangladesh |
| Tahmina Shirin | PhD | Institute of Epidemiology, Disease Control and Research (IEDCR) | [tahmina.shirin14@gmail.com](mailto:tahmina.shirin14@gmail.com) | Data collection | Bangladesh |
| Mahbubur Rahman | MPH | Institute of Epidemiology, Disease Control and Research (IEDCR) | [dr_mahbub@yahoo.com](mailto:dr_mahbub@yahoo.com) | Data collection | Bangladesh |
| Joseph Oundo | PhD | 1) London School of Hygiene & Tropical Medicine, United Kingdom, 2) College of Health and Medical Sciences, Haramaya University, Harar, Ethiopia | [joseph.oundo@lshtm.ac.uk](mailto:joseph.oundo@lshtm.ac.uk) | Data collection | Ethiopia |
| Alexander M. Ibrahim | MD | College of Health and Medical Sciences at Haramaya University | [alexandermi2002@yahoo.com](mailto:alexandermi2002@yahoo.com) | Data collection | Ethiopia |
| Fikremelekot Temesgen | MD | Addis Ababa University | [fikremelekot@gmail.com](mailto:fikremelekot@gmail.com) | Data collection | Ethiopia |
| Tadesse Gure | MD | College of Health and Medical Sciences at Haramaya University | [tadebuna@yahoo.com](mailto:tadebuna@yahoo.com) | Data collection | Ethiopia |
| Addisu Alemu | MD | College of Health and Medical Sciences at Haramaya University | [alemuadisu789@gmail.com](mailto:alemuadisu789@gmail.com) | Data collection | Ethiopia |
| Melisachew Mulatu Yeshi | MD | Ayder Specialized Comprehensive Hospital at Mekelle University | [melejose@yahoo.com](mailto:melejose@yahoo.com) | Data collection | Ethiopia |
| Mahlet Abayneh Gizaw | MD | St. Paul’s Hospital Millennium Medical College in Addis Ababa, Ethiopia | [mahletabayneh5@gmail.com](mailto:mahletabayneh5@gmail.com) | Data collection | Ethiopia |
| Stian MS Orlien | PhD | London School of Hygiene & Tropical Medicine | [stian@orlien.no](mailto:stian@orlien.no) | Data collection | Ethiopia |
| Solomon Ali | PhD | National Data Management Centre at the Ethiopian Public Health Institute | [solali2005@gmail.com](mailto:solali2005@gmail.com) | Data collection | Ethiopia |
| Kitiezo Aggrey Igunza^1^ | BSc | Maseno University, Kenya | [aigunza@kemri.go.ke](mailto:aigunza@kemri.go.ke) | Data collection | Kenya |
| Peter Otieno | MA | The University of Nairobi, Kenya | [POOtieno@kemri.go.ke](mailto:POOtieno@kemri.go.ke) | Data collection | Kenya |
| Peter Nyamthimba Onyango | MA | The University of Nairobi, Kenya | [nonyango@kemri.go.ke](mailto:nonyango@kemri.go.ke) | Data collection | Kenya |
| Janet Agaya | MPH | Maseno University, Kenya | [JAgaya@kemri.go.ke](mailto:JAgaya@kemri.go.ke) | Data collection | Kenya |
| Richard Oliech | Diploma in lab sciences | Kenya Polytechnic | [ROliech@kemri.go.ke](mailto:ROliech@kemri.go.ke) | Data collection | Kenya |
| Joyce Akinyi Were | MSc | University of Nairobi, Kenya | [JwereAkinyi@kemri.go.ke](mailto:JwereAkinyi@kemri.go.ke) | Data collection | Kenya |
| Dickson Gethi | BSc | University of Nairobi, Kenya | [DGethi@kemri.go.ke](mailto:DGethi@kemri.go.ke) | Data collection | Kenya |
| George Aol | MA | Great Lakes University of Kisumu, Kenya | [GAol@kemri.go.ke](mailto:GAol@kemri.go.ke) | Data collection | Kenya |
| Thomas Misore | MA | The University of Nairobi, Kenya | [TMisore@kemri.go.ke](mailto:TMisore@kemri.go.ke) | Data collection | Kenya |
| Harun Owuor | MSc | Jaramogi Oginga Odinga University of Science and Technology, Kenya | [HOwuor@kemri.go.ke](mailto:HOwuor@kemri.go.ke) | Data collection | Kenya |
| Christopher Muga | BSc | Jaramogi Oginga Odinga University of Science and Technology, Kenya | [CMugah@kemri.go.ke](mailto:CMugah@kemri.go.ke) | Data collection | Kenya |
| Bernard Oluoch | Diploma in Clinical Medicine & Surgery | Kenya Medical Training Institute, Nyeri, Kenya | [BOluoch@kemri.go.ke](mailto:BOluoch@kemri.go.ke) | Data collection | Kenya |
| Christine Ochola | Diploma in Clinical Medicine & Surgery | Kenya Medical Training Institute, Nyeri, Kenya | [COchola@kemri.go.ke](mailto:COchola@kemri.go.ke) | Data collection | Kenya |
| Sharon M. Tennant | PhD | University of Maryland School of Medicine, Baltimore, Maryland, USA | [stennant@som.umaryland.edu](mailto:stennant@som.umaryland.edu) | Data collection | Mali |
| Carol L. Greene | MD | University of Maryland School of Medicine, Baltimore, Maryland, USA | [carol.greene@som.umaryland.edu](mailto:carol.greene@som.umaryland.edu) | Data collection | Mali |
| Ashka Mehta | MPH | Department of Pediatrics, Center for Vaccine Development and Global Health, University of Maryland School of Medicine, Baltimore, Maryland, USA | [amehta@som.umaryland.edu](mailto:amehta@som.umaryland.edu) | Data collection | Mali |
| J. Kristie Johnson | PhD | University of Maryland School of Medicine, Baltimore, Maryland, USA | [jkjohnson@som.umaryland.edu](mailto:jkjohnson@som.umaryland.edu) | Data collection | Mali |
| Brigitte Gaume | PhD | Center for Vaccine Development and Global Health, University of Maryland School of Medicine, Baltimore, Maryland, USA | bgaume@som.umaryland.edu | Data collection | Mali |
| Adama Mamby Keita | MD | Centre pour le Développement des Vaccins (CVD-Mali), Ministère de la Santé | [akeita@cvd-mali.org](mailto:akeita@cvd-mali.org) | Data collection | Mali |
| Rima Koka | MD | University of Maryland School of Medicine, Baltimore, Maryland, USA | [mkoka@umm.edu](mailto:mkoka@umm.edu) | Data collection | Mali |
| Karen D. Fairchild | MD | University of Virginia | [kdf2n@hscmail.mcc.virginia.edu](mailto:kdf2n@hscmail.mcc.virginia.edu) | Data collection | Mali |
| Diakaridia Kone | MD | CSRef Commune I, Bamako, Mali | [diakvi@yahoo.fr](mailto:diakvi@yahoo.fr) | Data collection | Mali |
| Diakaridia Sidibe | MD | Centre pour le Développement des Vaccins (CVD-Mali), Ministère de la Santé | [diaksidibe72@gmail.com](mailto:diaksidibe72@gmail.com) | Data collection | Mali |
| Doh Sanogo | MD | Epidemiology Department CVD-Mali, Bamako, Mali | [sanogodoh@yahoo.fr](mailto:sanogodoh@yahoo.fr) | Data collection | Mali |
| Uma U. Onwuchekwa | MSc | Bioinformatics department, CVD-Mali, Bamako, Mali | [uonwuche@som.umaryland.edu](mailto:uonwuche@som.umaryland.edu) | Data collection | Mali |
| Nana Kourouma | MD, PHD | CVD-Mali, HGT, Bamako, Mali | [knana1000@yahoo.fr](mailto:knana1000@yahoo.fr) | Data collection | Mali |
| Seydou Sissoko | MD | CVD-Mali, HGT, Bamako, Mali | [seydinet7@yahoo.fr](mailto:seydinet7@yahoo.fr) | Data collection | Mali |
| Cheick Bougadari Traore | MD | CHU POINT G, Bamako Mali | [cheickbtraore@yahoo.fr](mailto:cheickbtraore@yahoo.fr) | Data collection | Mali |
| Jane Juma | Ms, HND in Biotechnology | CVD-Mali, HGT, Bamako, Mali | jjuma@cvd-mali.org | Data collection | Mali |
| Kounandji Diarra | MSc | CNAM/CVD-Mali, Bamako, Mali | diarrakounandji@yahoo.fr | Data collection | Mali |
| Awa Traore | MSc | CNAM/CVD-Mali, Bamako, Mali | atraore@cvd-mali.org | Data collection | Mali |
| Tiéman Diarra | PhD, Professor | Point-Sud, Bamako, Mali | jaraceman@yahoo.fr | Data collection | Mali |
| Kiranpreet Chawla | MD | Department of Obstetrics, Gynecology and Reproductive Sciences, University of Maryland School of Medicine, Baltimore, Maryland, USA | kchawla@som.umaryland.edu | Data collection | Mali |
| Tacilta Nhampossa |  |  | [tacilda.nhampossa@manhica.net](mailto:tacilda.nhampossa@manhica.net) | Data collection | Mozambique |
| Zara Manhique |  |  | [zaraonila@gmail.com](mailto:zaraonila@gmail.com) | Data collection | Mozambique |
| Sibone Mocumbi |  |  | [sibone.m@gmail.com](mailto:sibone.m@gmail.com) | Data collection | Mozambique |
| Clara Menéndez |  |  | [clara.menendez@isglobal.org](mailto:clara.menendez@isglobal.org) | Data collection | Mozambique |
| Khátia Munguambe |  | Centro de Investigacao em Saude de Manhica Eduardo Mondlane University, Faculty of Medicine, Community Health Department, Maputo, Mozambique | [khatia.munguambe@manhica.net](mailto:khatia.munguambe@manhica.net) | Data collection | Mozambique |
| Ariel Nhacolo |  | Centro de Investigação em Saúde de Manhiça [CISM] | [Ariel.Nhacolo@manhica.net](mailto:Ariel.Nhacolo@manhica.net) | Data collection | Mozambique |
| Maria Maixenchs |  | IS Global Hospital Clinic--Universitat de Barcelona, Spain Centro de Investigacao en Saude de Manhica (CISM), Manhica, Mozambique | [maria.maixenchs@isglobal.org](mailto:maria.maixenchs@isglobal.org) | Data collection | Mozambique |
| Andrew Moseray | MSc | Crown Agents | [andrew.moseray@crownagents.co.uk](mailto:andrew.moseray@crownagents.co.uk) | Data collection | Sierra Leone |
| Fatmata Bintu Tarawally | MSc | FOCUS 1000 | tfatmatabintu@gmail.com | Data collection | Sierra Leone |
| Martin Seppeh | BSc | FOCUS 1000 | martinmusaseppeh@gmail.com | Data collection | Sierra Leone |
| Ronald Mash | DrPH | Ministry of Health and Sanitation, Freetown, Sierra Leone | [naldoline@yahoo.com](mailto:naldoline@yahoo.com) | Data collection | Sierra Leone |
| Julius Ojulong | MD | Crown Agents | [julius.ojulong@crownagents.co.uk](mailto:julius.ojulong@crownagents.co.uk) | Data collection | Sierra Leone |
| Babatunde Duduyemi | FMCPath | University of Sierra Leone Teaching Hospital Complex, Freetown | [babsdudu@yahoo.com](mailto:babsdudu@yahoo.com) | Data collection | Sierra Leone |
| James Bunn | MD | Human Development Team, British High Commission, Freetown | [bunnj@who.int](mailto:bunnj@who.int) | Data collection | Sierra Leone |
| Alim Swaray-Deen | FWACS - Ob/Gyn  (Fellowship of the West African College of Surgeons) | University of Sierra Leone Teaching Hospital Complex, Freetown | [asdeen8@gmail.com](mailto:asdeen8@gmail.com) | Data collection | Sierra Leone |
| Joseph Bangura | MPH | Ministry of Health and Sanitation, Freetown, Sierra Leone | [bangsylj@yahoo.com](mailto:bangsylj@yahoo.com) | Data collection | Sierra Leone |
| Amara Jambai | MSc | Ministry of Health and Sanitation, Freetown, Sierra Leone | [amarajambai@yahoo.com](mailto:amarajambai@yahoo.com) | Data collection | Sierra Leone |
| Margaret Mannah | MPH | Ministry of Health and Sanitation, Freetown, Sierra Leone | [mtmannah@yahoo.co.uk](mailto:mtmannah@yahoo.co.uk) | Data collection | Sierra Leone |
| Okokon Ita | FMCPath - Medical Microbiology and Parasitology | University of Calabar Teaching Hospital, Nigeria | [itaokokonim@gmail.com](mailto:itaokokonim@gmail.com) | Data collection | Sierra Leone |
| Cornell Chukwuegbo | FMCPath - Anatomical Pathologist | Federal Medical Center/PathConsult Diagnostics Ltd. Umuahia, Nigeria | [chukwuegbocc@gmail.com](mailto:chukwuegbocc@gmail.com) | Data collection | Sierra Leone |
| Sulaiman Sannoh | MD | St. Luke's University Health Network, Easton, Pennsylvania, USA | [sannohsu@gmail.com](mailto:sannohsu@gmail.com) | Data collection | Sierra Leone |
| Princewill Nwajiobi | FMCPath – Medical Microbiology | National Hospital, Abuja, Nigeria | [princewill101@yahoo.com](mailto:princewill101@yahoo.com) | Data collection | Sierra Leone |
| Dickens Kowuor | MSc | Crown Agents | [dickens.kowuor@crownagents.co.uk](mailto:dickens.kowuor@crownagents.co.uk) | Data collection | Sierra Leone |
| Erick Kaluma | MPH | Crown Agents | [erick.kaluma@crownagents.co.uk](mailto:erick.kaluma@crownagents.co.uk) | Data collection | Sierra Leone |
| Oluseyi Balogun | MHM | Crown Agents | [oluseyi.balogun@crownagents.co.uk](mailto:oluseyi.balogun@crownagents.co.uk) | Data collection | Sierra Leone |
| Carrie Jo Cain | RN | World Hope International, Makeni, Sierra Leone | [carriejocain@worldhope.org](mailto:carriejocain@worldhope.org) | Data collection | Sierra Leone |
| Solomon Samura | BSc | World Hope International, Makeni, Sierra Leone | [solomon.samura@worldhope.org](mailto:solomon.samura@worldhope.org) | Data collection | Sierra Leone |
| Samuel Pratt | MPH | FOCUS 1000 | [samuel.pratt13@yahoo.com](mailto:samuel.pratt13@yahoo.com) | Data collection | Sierra Leone |
| Francis Moses | Master of Medicine | Ministry of Health and Sanitation, Freetown, Sierra Leone | [franqoline@gmail.com](mailto:franqoline@gmail.com) | Data collection | Sierra Leone |
| Tom Sesay |  | Ministry of Health and Sanitation, Freetown, Sierra Leone | [tommahunsesay@gmail.com](mailto:tommahunsesay@gmail.com) | Data collection | Sierra Leone |
| James Squire | MPhil Applied Epidemiology and Disease Control | Ministry of Health and Sanitation, Freetown, Sierra Leone | [jmssuire@yahoo.com](mailto:jmssuire@yahoo.com) | Data collection | Sierra Leone |
| Joseph Kamanda Sesay |  | Ministry of Health and Sanitation, Freetown, Sierra Leone | j.kasay@yahoo.com | Data collection | Sierra Leone |
| Osman Kaykay | MMed in Obstetrics and Gynaecology | Ministry of Health and Sanitation, Freetown, Sierra Leone | kalkulux@gmail.com | Data collection | Sierra Leone |
| Binyam Halu | MPH | WHO | [mahawadumbuyaconteh@yahoo.com](mailto:mahawadumbuyaconteh@yahoo.com) | Data collection | Sierra Leone |
| Hailemariam Legesse | Postgraduate Diploma in Paediatrics and Child health | UNICEF | [hlegesse@unicef.org](mailto:hlegesse@unicef.org) | Data collection | Sierra Leone |
| Francis Smart |  | Ministry of Health and Sanitation, Freetown, Sierra Leone | [drfsmart@mail.com](mailto:drfsmart@mail.com) | Data collection | Sierra Leone |
| Sartie Kenneh |  | Ministry of Health and Sanitation, Freetown, Sierra Leone | [sartiekenneh@gmail.com](mailto:sartiekenneh@gmail.com) | Data collection | Sierra Leone |
| Soter Ameh | PhD | Crown Agents | sotersunday.ameh@crownagents.co.uk | Data collection | Sierra Leone |
| Jana Ritter | DVM | Infectious Diseases Pathology Branch, Division of High-Consequence Pathogens and Pathology, National Center for emerging and Zoonotic Infectious Diseases, Centers for Disease Control and Prevention, Atlanta, US | [vtr0@cdc.gov](mailto:vtr0@cdc.gov) | Data collection | PO / CPL |
| Tais Wilson | DVM | Centers for Disease Control and Prevention | [qdh6@cdc.gov](mailto:qdh6@cdc.gov) | Data collection | PO / CPL |
| Jonas Winchell | PhD | Respiratory Diseases Branch, Division of Bacterial Diseases, National Center for Immunization and Respiratory Diseases, Centers for Disease Control and Prevention, Atlanta, US | [zdx2@cdc.gov](mailto:zdx2@cdc.gov) | Data collection | PO / TAC |
| Jakob Witherbee | BS | Centers for Disease Control and Prevention | [oqv3@cdc.gov](mailto:oqv3@cdc.gov) | Data collection | PO / TAC |
| Mischka Garel | MPH | Emory Global Health Institute, Emory University, Atlanta, Georgia | [mgarel@emory.edu](mailto:mgarel@emory.edu) | Data collection | PO / 1599 |
| Navit T. Salzberg | MPH | Emory Global Health Institute, Emory University, Atlanta, Georgia | [nsalzberg@taskforce.org](mailto:nsalzberg@taskforce.org) | Data collection | PO / 1599 |
| Jeffrey P. Koplan | MD | Emory Global Health Institute, Emory University, Atlanta, Georgia, USA | [jkoplan@emory.edu](mailto:jkoplan@emory.edu) | Data collection | PO / 1599 |
| Kyu Han Lee | PhD | Emory Global Health Institute, Emory University, Atlanta, Georgia, USA | [klee75@emory.edu](mailto:klee75@emory.edu) | Data collection | PO |
| Roosecelis Martines |  |  | [xgn7@cdc.gov](mailto:xgn7@cdc.gov) | Data collection | CPL |
| Shamta Warang |  |  | [rxn4@cdc.gov](mailto:rxn4@cdc.gov) | Data collection | CPL |
| Maureen Diaz |  |  | [iqs5@cdc.gov](mailto:iqs5@cdc.gov) | Data collection | TAC |
| Jessica Waller |  |  | [kbu9@cdc.gov](mailto:kbu9@cdc.gov) | Data collection | TAC |
| Shailesh Nair | MPH | Public Health Informatics Institute, The Task Force for Global Health, Atlanta, Georgia | [snair@taskforce.org](mailto:snair@taskforce.org) | Data collection | PO |
| Lucy Liu | MBA | Public Health Informatics Institute at the Task Force for Global Health in Atlanta, Georgia, USA | [lliu@taskforce.org](mailto:lliu@taskforce.org) | Data collection | PO |
| Courtney Bursuc | MPH | Emory Global Health Institute, Emory University, Atlanta, GA, USA | [courtney.bursuc@emory.edu](mailto:courtney.bursuc@emory.edu) | Data collection | PO |
| Kristin LaHatte | MA | Emory Global Health Institute, Emory University, Atlanta, GA, USA | [Kristin.lahatte@emory.edu](mailto:Kristin.lahatte@emory.edu) | Data collection | PO |
| Sarah Raymer | BA | Emory Global Health Institute, Emory University, Atlanta, GA, USA | [sarah.raymer@emory.edu](mailto:sarah.raymer@emory.edu) | Data collection | PO |
| John Blevins | ThD | Emory Global Health Institute, Emory University, Atlanta, GA, USA | [jblevin@emory.edu](mailto:jblevin@emory.edu) | Data collection | PO |
| Solveig Argeseanu | PhD | Emory Global Health Institute, Emory University, Atlanta, GA, USA | [sargese@emory.edu](mailto:sargese@emory.edu) | Data collection | PO |
| Kurt Vyas | PhD | Emory Global Health Institute, Emory University, Atlanta, GA, USA | [kartavya.jayant.vyas@emory.edu](mailto:kartavya.jayant.vyas@emory.edu) | Data collection | PO |
| Manu Bhandari | MPH | Emory Global Health Institute, Emory University, Atlanta, GA, USA | [manu.simriti.bhandari@emory.edu](mailto:manu.simriti.bhandari@emory.edu) | Data collection | PO |
| Fatima Solomon | MD | South African Medical Research Council Vaccines and Infectious Diseases Analytics Research Unit, University of the Witwatersrand, Johannesburg, South Africa | [fatima.solomon@wits-vida.org](mailto:fatima.solomon@wits-vida.org) | MITS | South Africa |
| Gillian Sorour | MD | Wits Health Consortium | [gilllian.sorour@wits.ac.za](mailto:gilllian.sorour@wits.ac.za) | MITS | South Africa |
| Hennie Lombaard | MD | University of Witwatersrand, Johannesburg, South Africa | [hennie.lombaard73@gmail.com](mailto:hennie.lombaard73@gmail.com) | MITS | South Africa |
| Jeannette Wadula | MD | National Health Laboratory Service, Department of Microbiology and Infectious Diseases, School of Pathology, University of the Witwatersrand, Faculty of Health Sciences, Johannesburg, South Africa | [jeannette.wadula@wits.ac.za](mailto:jeannette.wadula@wits.ac.za) | MITS | South Africa |
| Karen Petersen | MD | Department of Paediatrics, Chris Hani Baragwanath Academic Hospital, Faculty of Health Sciences, University of the Witwatersrand, Johannesburg, South Africa | [karen.petersen@wits.ac.za](mailto:karen.petersen@wits.ac.za) | MITS | South Africa |
| Martin Hale | MD | National Health Laboratory Service, Department of Anatomical Pathology, School of Pathology, University of the Witwatersrand, Faculty of Health Sciences, Johannesburg, South Africa | [martin.hale@nhls.ac.za](mailto:martin.hale@nhls.ac.za) | MITS | South Africa |
| Nelesh P. Govender | MD | National Institute for Communicable Diseases (NICD), Johannesburg, South Africa | [neleshg@nicd.ac.za](mailto:neleshg@nicd.ac.za) | MITS | South Africa |
| Peter J. Swart | MD | National Health for Laboratory Service in South Africa | [peter.swart@nhls.ac.za](mailto:peter.swart@nhls.ac.za) | MITS | South Africa |
| Sanjay G. Lala | MD | Department of Paediatrics and Perinatal HIV Research Unit, Chris Hani Baragwanath Academic Hospital, Faculty of Health Sciences, University of the Witwatersrand, Johannesburg, South Africa | [sanjay.lala@wits.ac.za](mailto:sanjay.lala@wits.ac.za) | MITS | South Africa |
| Sithembiso Velaphi | PhD | Department of Pediatrics, Chris Hani Baragwanath Academic Hospital, School of Clinical Medicine, Faculty of Health Sciences, University of the Witwatersrand, Johannesburg, South Africa | [sithembiso.velaphi@wits.ac.za](mailto:sithembiso.velaphi@wits.ac.za) | MITS | South Africa |
| Richard Chawana | PhD | South African Medical Research Council Vaccines and Infectious Diseases Analytics Research Unit, University of the Witwatersrand, Johannesburg, South Africa | [richardc@biovac.co.za](mailto:richardc@biovac.co.za) | MITS | South Africa |
| Yasmin Adam | MD | 1) Department of Obstetrics & Gynaecology, Chris Hani Baragwanath Academic Hospital, School of Clinical Medicine, 2) Faculty of Health Sciences, Univeristy of the Witwatersrand, Faculty Health Sciences, Johannesburg, South Africa | [yasminadam@gmail.com](mailto:yasminadam@gmail.com) | MITS | South Africa |
| Amy Wise | MSc | South African Medical Research Council Vaccines and Infectious Diseases Analytics Research Unit, University of the Witwatersrand, Johannesburg, South Africa | [amyjulietwise@yahoo.co.uk](mailto:amyjulietwise@yahoo.co.uk) | MITS | South Africa |
| Nellie Myburgh | PhD | South African Medical Research Council Vaccines and Infectious Diseases Analytics Research Unit, University of the Witwatersrand, Johannesburg, South Africa | [nellie.myburgh@wits-vida.org](mailto:nellie.myburgh@wits-vida.org) | SBS | South Africa |
| Lunghile Shivambo |  | South African Medical Research Council Vaccines and Infectious Diseases Analytics Research Unit, University of the Witwatersrand, Johannesburg, South Africa | [lunghile.shivambo@wits-vida.org;](mailto:lunghile.shivambo@wits-vida.org) | SBS | South Africa |
| Lerato Ntsie |  | South African Medical Research Council Vaccines and Infectious Diseases Analytics Research Unit, University of the Witwatersrand, Johannesburg, South Africa | [lerato.ntsie@wits-vida.org;](mailto:lerato.ntsie@wits-vida.org) | SBS | South Africa |
| Thabisile Qwabi |  | South African Medical Research Council Vaccines and Infectious Diseases Analytics Research Unit, University of the Witwatersrand, Johannesburg, South Africa | [thabisile.qwabi@wits-vida.org](mailto:thabisile.qwabi@wits-vida.org) | SBS | South Africa |
| Sana Mahtab | PhD | South African Medical Research Council Vaccines and Infectious Diseases Analytics Research Unit, University of the Witwatersrand, Johannesburg, South Africa | [sana.mahtab@wits-vida.org](mailto:sana.mahtab@wits-vida.org) | All | South Africa |
| Jeanie du Toit | MD | South African Medical Research Council Vaccines and Infectious Diseases Analytics Research Unit, University of the Witwatersrand, Johannesburg, South Africa | [jeanie.dutoit@wits-vida.org](mailto:jeanie.dutoit@wits-vida.org) |  | South Africa |
| Megan Dempster | MD | South African Medical Research Council Vaccines and Infectious Diseases Analytics Research Unit, University of the Witwatersrand, Johannesburg, South Africa | [megan.dempster@wits-vida.org](mailto:megan.dempster@wits-vida.org) |  | South Africa |
| Siobhan Johnstone | PhD | South African Medical Research Council Vaccines and Infectious Diseases Analytics Research Unit, University of the Witwatersrand, Johannesburg, South Africa | [siobhan.johnstone@wits-vida.org](mailto:siobhan.johnstone@wits-vida.org) | All | South Africa |
| Ziyaad Dangor | PhD | South African Medical Research Council Vaccines and Infectious Diseases Analytics Research Unit, University of the Witwatersrand, Johannesburg, South Africa | [ziyaad.dangor@wits-vida.org](mailto:ziyaad.dangor@wits-vida.org) | All | South Africa |
| Shabir Madhi | PhD | South African Medical Research Council Vaccines and Infectious Diseases Analytics Research Unit, University of the Witwatersrand, Johannesburg, South Africa | [shabir.madhi@wits-vida.org](mailto:shabir.madhi@wits-vida.org) | All | South Africa |
| Tanya Ruder | MD | Division of Community Paediatrics, Department of Paediatrics and Child Health | [tanya.ruder@wits.ac.za](mailto:tanya.ruder@wits.ac.za) | MITS (Comm. Paeds) | South Africa |
| Vuyelwa Baba | MD | Gauteng Department of Health |  | MITS (O&G) | South Africa |
| Lesego Mothibi | MD | NHLS | [lesego.mothibi@wits.ac.za](mailto:lesego.mothibi@wits.ac.za) | MITS (Micro) | South Africa |
| Prenika Jaglal | MD | Clinical Microbiology and Infectious Diseases, Chris Hani Baragwanath Academic Hospital, University of the Witwatersrand/National Health Laboratory Services | [prenika.jaglal@wits.ac.za](mailto:prenika.jaglal@wits.ac.za) | MITS (Micro) | South Africa |
| Firdose Nakwa | MD | Paediatrics & Child Health (Chris Hani Baragwanath Hospital) | [firdose.nakwa@wits.ac.za](mailto:firdose.nakwa@wits.ac.za) | Neonates | South Africa |
| Kimberleigh Storath | CA | South African Medical Research Council Vaccines and Infectious Diseases Analytics Research Unit, University of the Witwatersrand, Johannesburg, South Africa | [kimberleigh.storath@wits-vida.org](mailto:kimberleigh.storath@wits-vida.org) | MITS | South Africa |
| Michelle Groome | PhD | South African Medical Research Council Vaccines and Infectious Diseases Analytics Research Unit, University of the Witwatersrand, Johannesburg, South Africa | [michelle.groome@wits-vida.org](mailto:michelle.groome@wits-vida.org) | Pregnancy surveillance | South Africa |
| Vicky Baillie | PhD | South African Medical Research Council Vaccines and Infectious Diseases Analytics Research Unit, University of the Witwatersrand, Johannesburg, South Africa | [vicky.baillie@wits-vida.org](mailto:vicky.baillie@wits-vida.org) | Lab | South Africa |
| Takwanisa Machemedze | PhD | South African Medical Research Council Vaccines and Infectious Diseases Analytics Research Unit, University of the Witwatersrand, Johannesburg, South Africa | [takwanisa.machemedze@wits-vida.org](mailto:takwanisa.machemedze@wits-vida.org) | HDSS | South Africa |
| Alane Izu | PhD | South African Medical Research Council Vaccines and Infectious Diseases Analytics Research Unit, University of the Witwatersrand, Johannesburg, South Africa | [alane.izu@wits-vida.org](mailto:alane.izu@wits-vida.org) | Data | South Africa |
| Courtney Olwagen | PhD | South African Medical Research Council Vaccines and Infectious Diseases Analytics Research Unit, University of the Witwatersrand, Johannesburg, South Africa | [courtney.olwagen@wits-vida.org](mailto:courtney.olwagen@wits-vida.org) | Lab | South Africa |
| Bongani Ntimani | Nursing | South African Medical Research Council Vaccines and Infectious Diseases Analytics Research Unit, University of the Witwatersrand, Johannesburg, South Africa | [bongani.ntimani@wits-vida.org](mailto:bongani.ntimani@wits-vida.org) | Pregnancy surveillance | South Africa |
| Marguerite Hall | MD | South African Medical Research Council Vaccines and Infectious Diseases Analytics Research Unit, University of the Witwatersrand, Johannesburg, South Africa | [marguerite.hall@wits-vida.org](mailto:marguerite.hall@wits-vida.org) | MITS | South Africa |
| Shaakeera Holland | MD | Forensic Pathology Chris Hani Baragwanath Hospital | [skakeera.holland@wits.ac.za](mailto:skakeera.holland@wits.ac.za) | MITS (Genetics) | South Africa |
| Zane Lombard | PhD | Human Genetics, University of the Witwatersrand | [zane.lombard@wits.ac.za](mailto:zane.lombard@wits.ac.za) | MITS (Genetics) | South Africa |
| Michael Urban | PhD | Human Genetics, University of the Witwatersrand | [michael.urban@wits.ac.za](mailto:michael.urban@wits.ac.za) | MITS (Genetics) | South Africa |
| Amanda Krause | PhD | Human Genetics, University of the Witwatersrand | [amanda.krause@wits.ac.za](mailto:amanda.krause@wits.ac.za) | MITS (Genetics) | South Africa |
| Xumani Ndlovu |  | South African Medical Research Council Vaccines and Infectious Diseases Analytics Research Unit, University of the Witwatersrand, Johannesburg, South Africa | [xumani.ndlovu@wits-vida.org](mailto:xumani.ndlovu@wits-vida.org) | Data | South Africa |
| Selamo Tloubatla |  | South African Medical Research Council Vaccines and Infectious Diseases Analytics Research Unit, University of the Witwatersrand, Johannesburg, South Africa | [selamo.tloubatla@wits-vida.org](mailto:selamo.tloubatla@wits-vida.org) | Data | South Africa |
| Marius Laubscher | MSc | South African Medical Research Council Vaccines and Infectious Diseases Analytics Research Unit, University of the Witwatersrand, Johannesburg, South Africa | [marius.laubscher@wits-vida.org](mailto:marius.laubscher@wits-vida.org) | Lab | South Africa |
| Jeanine du Plessis | MD | South African Medical Research Council Vaccines and Infectious Diseases Analytics Research Unit, University of the Witwatersrand, Johannesburg, South Africa | [jeanine.duplessis@wits-vida.org](mailto:jeanine.duplessis@wits-vida.org) | Lab | South Africa |
